# Bibliometric analysis of ferroptosis: a comprehensive evaluation of its contribution to lung cancer

**DOI:** 10.3389/fgene.2024.1449491

**Published:** 2025-01-29

**Authors:** Feifei Yao, Feng Guo, Chuanzhong Sun, Erdong Wang, Hang Wang, Na Li

**Affiliations:** ^1^ Department of Respiratory and Critical Care Medicine, Suzhou Hospital of Anhui Medical University, Suzhou, China; ^2^ Department of Respiratory Medicine, Yiwu Chouzhou Hospital, Yiwu, China

**Keywords:** ferroptosis, lung cancer, CiteSpace, bibliometric analysis, knowledge mapping

## Abstract

**Objectives:**

This study employs bibliometric analysis to track evolution and identify trends of key topics in ferroptosis within the context of lung cancer. By identifying emerging research areas, our aim is to provide valuable insights and directions for researchers in this field.

**Methods:**

Relevant papers and reviews on ferroptosis in lung cancer were retrieved from the Web of Science Core Collection database on 5 February 2024. Bibliometric analysis was conducted using CiteSpace 6.2.R3, VOSviewer 1.6.20, R 4.3.0, Bibliometric and Microsoft Excel 2019.

**Results:**

From 2015 to 2020, publications related to ferroptosis in lung cancer were sparse but showed a steady increase. Post-2020, there has been a significant surge, with a 6.4-fold increase observed by 2023. Overall, authors from 4,033 institutions across 42 countries/regions contributed 606 papers published in 262 academic journals. China emerged as the leading contributor, while the United States maintained dominance. Lifang Ma was the most prolific author, with DIXON SJ, YANG WS, and STOCKWELL BR being the most frequently co-cited. Effective communication and collaboration among scholars are lacking. Key journals include Frontiers in Pharmacology for publication output, and Nature and Cell for citation frequency. Research focuses on molecular mechanisms of ferroptosis, including endoplasmic reticulum stress, tumor microenvironment, and autophagy. Therapeutic targets like GPX4, SLC7A11, P53, FSP1, Nrf2, LSH, STYK1/NOK, and ACSL4 are prominent. Traditional Chinese medicine also shows clinical value in ferroptosis research.

**Conclusion:**

Ferroptosis, as a promising research avenue with significant clinical applications in lung cancer, continues to undergo rapid development. The study of iron death in lung cancer will remain a critical research focus in the future.

## 1 Introduction

Cancer poses a significant health risk and remains a primary cause of human mortality (Siegel et al., 2023). Among the array of cancer types, lung cancer stands as the second most commonly diagnosed and the foremost cause of cancer-related deaths, contributing to around 18% of all such fatalities (Sung et al., 2021). Despite advancements in conventional therapies, along with molecular targeted treatments and immunotherapy, outcomes for patients with advanced-stage lung cancer have proven unsatisfactory (Zukotynski et al., 2021). Hence, there is a pressing need for deeper exploration into the mechanisms governing the onset and advancement of lung cancer, alongside the quest for innovative and effective therapeutic approaches.

Programmed cell death pathways, such as apoptosis, necroptosis, and pyroptosis, have long been recognized as critical regulators of cancer biology. Recently, novel regulated cell death mechanisms, including cuproptosis and disulfidptosis, have been identified, offering fresh insights into tumor progression and therapeutic strategies. Cuproptosis, a copper-dependent form of cell death, has been implicated in cancer progression, with pan-cancer analyses revealing that mutations in cuproptosis-associated genes (e.g., ATP7A, ATP7B, LIAS) correlate with survival outcomes and immune microenvironment modulation in multiple cancer types, including kidney and uterine cancers (Liu and Tang, 2022a; Liu and Tang, 2022b; Liu, 2023). Similarly, disulfidptosis, a disulfide stress-mediated cell death mechanism, has been linked to aberrant cytoskeletal dynamics and associated with prognosis in cancers such as mesothelioma and lower-grade glioma (Liu et al., 2023a; Liu and Tang, 2023). Both pathways highlight the critical role of regulated cell death in influencing cancer development, immune infiltration, and therapeutic sensitivity, underscoring their potential as diagnostic and prognostic biomarkers.

Among these, ferroptosis, introduced by Dixon et al., in 2012, has garnered significant attention due to its unique iron-dependent mechanism involving the accumulation of lipid peroxides and glutathione peroxidase (GPX) inhibition, leading to oxidative membrane damage (Dixon et al., 2012; Kinowaki et al., 2021). Subsequent studies have demonstrated that ferroptosis plays a crucial role in lung cancer progression, with its induction emerging as a promising therapeutic strategy (Chai et al., 2021; Liu et al., 2021). Furthermore, research has revealed that ferroptosis not only impedes tumor growth but also enhances the efficacy of chemotherapy, targeted therapy, and immunotherapy (Guo et al., 2018; Sun et al., 2016). Recent studies have emphasized the therapeutic potential of targeting ferroptosis-related pathways, such as system Xc− inhibition and GPX4 degradation, for overcoming therapy resistance and suppressing metastasis in lung cancer (Xia et al., 2019; Kim et al., 2023; Fan et al., 2025). Additionally, investigations into nanomaterial-based ferroptosis inducers have shown promise in preclinical cancer treatment models, paving the way for innovative therapeutic strategies (Hsieh et al., 2021; Shu et al., 2024). It should be noted that there are currently drugs in development targeting ferroptosis-related mechanisms, such as small-molecule compounds that regulate iron metabolism and enhance GPX4 activity, which may offer new avenues for treatment (Ding et al., 2021; Ye et al., 2022; Zhang et al., 2022a). Despite these advances, the precise mechanisms governing ferroptosis remain incompletely understood, necessitating further exploration into its intricate regulatory network and therapeutic implications. Given the rapidly increasing interest in ferroptosis and its therapeutic potential in lung cancer, there is a pressing need to systematically evaluate the current state of research and identify emerging trends. Several reviews have synthesized research in this area from various perspectives (Wu et al., 2021; Zou et al., 2021; Xing et al., 2023; Zuo et al., 2022; Niu et al., 2022). However, to the best of our knowledge, no comprehensive bibliometric analysis has been conducted on ferroptosis research in lung cancer. Bibliometric methods, unlike traditional reviews, enable both qualitative and quantitative assessments of the contributions and collaborations of authors, institutions, and countries, providing insights into current research hotspots and future directions (Chen and Song, 2019; Zhang et al., 2021a).

Therefore, this paper employs bibliometric analysis to quantitatively evaluate the characteristics and trends of research in the field of ferroptosis in lung cancer from 2015 to 2024. Through this approach, we aim to identify research hotspots and emerging trends, offering guidance for future studies in this rapidly evolving field.

## 2 Materials and methods

### 2.1 Data sources and search strategy

The Web of Science Core Collection (WoSCC) database is widely utilized in bibliometrics. The data were extracted from the WoSCC database on 5 February 2024. The search terms included: [TS = (“lung cancer” OR “lung carcinoma” OR “non-small cell lung cancer” OR “small cell lung cancer”)] AND [TS = ferroptosis]. The subject headings and free words used in the search were: [TS = (“lung cancer,” “lung carcinoma,” “non-small cell lung cancer,” “small cell lung cancer,” “lung neoplasm,” “lung tumor,” “ferroptosis,” “iron death,” “oxidative stress”, “cell death,” “iron metabolism”)]. The search indices comprised Science Citation Index Expanded (SCI-Expanded), Social Sciences Citation Index (SSCI), Arts Humanities Citation Index (AHCI), Conference Proceedings Citation Index-Science (CPCI-S), Conference Proceedings Citation Index-Social Science & Humanities (CPCI-SSH), Book Citation Index Science (BKCI-S), Book Citation Index -Social Sciences Humanities (BKCI-SSH), and Emerging Sources Citation Index (ESCI). We restricted our search to English-language literature and limited the document types to reviews and articles. Search results were downloaded in “Full Record and Cited References” and “Plain Text” formats.

### 2.2 Literature quantity

**FIGURE 1:**
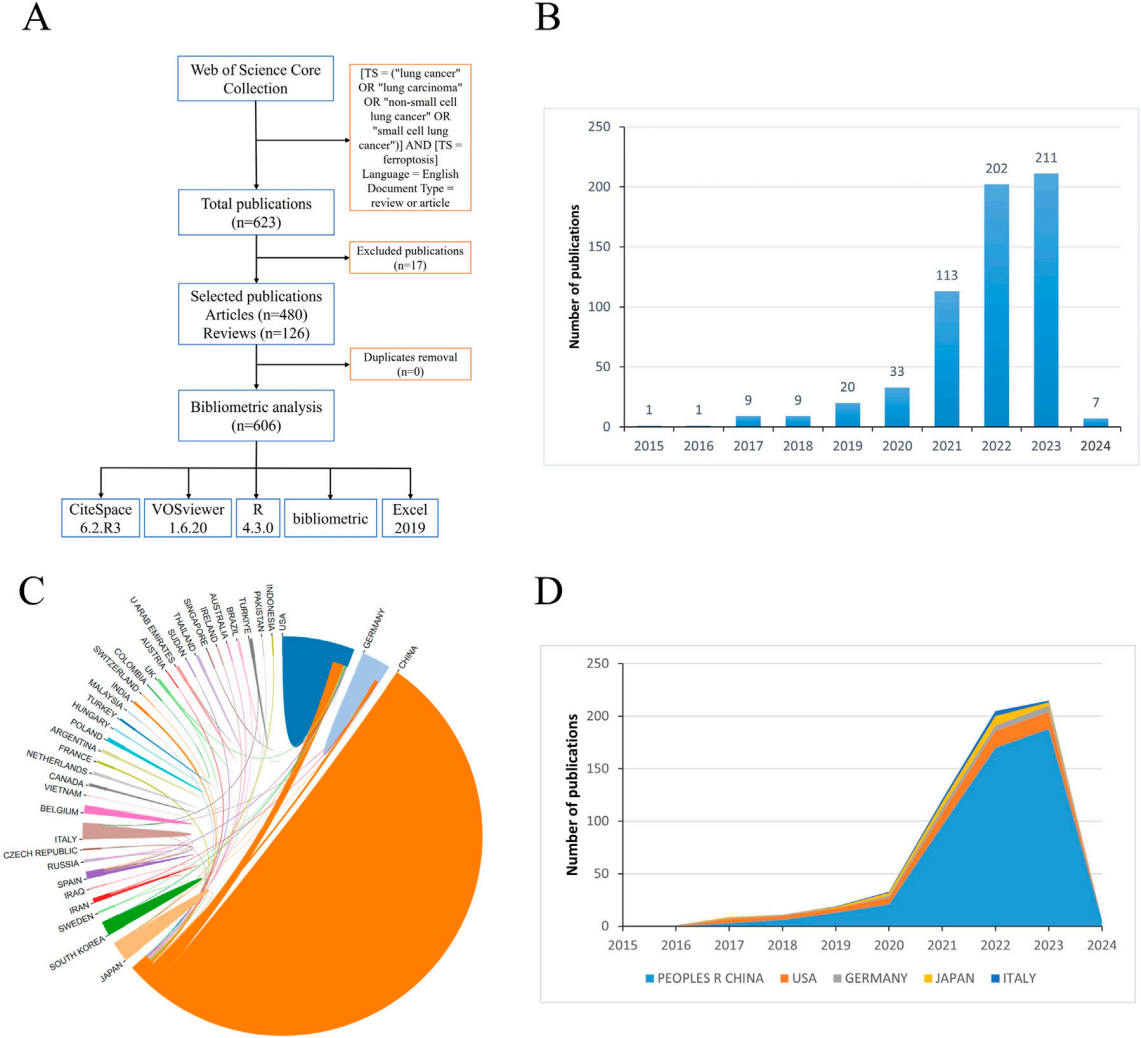
Flow chart of data collection, trends in publications and international collaboration in the field of ferroptosis in lung cancer **(A)**. Flow chart of data collection; **(B)** Bar chart illustrating the annual publication trends in the field of ferroptosis in lung cancer; **(C)** Pie chart depicting international collaboration in the field of ferroptosis in lung cancer; **(D)** Stacked area chart showing the annual publication volume of the top 5 countries in the field of ferroptosis in lung cancer.

From 1 January 2015, to 5 February 2024, our comprehensive search yielded a total of 623 relevant references. After excluding ineligible and duplicate articles, we retained 606 articles and reviews focusing on airway remodeling for analysis (Figure 1A).

### 2.3 Visualization and statistical analyses

Bibliometric analysis and visualization were performed using CiteSpace 6.2.R3, VOSviewer 1.6.20, R 4.3.0, bibliometric, and Microsoft Excel 2019. Initially, we implemented data cleaning procedures, consolidating synonymous terms, and removing irrelevant vocabulary. Subsequently, CiteSpace was employed to examine various facets, including the co-occurrence of institutions and authors, dual-map representations of journals, reference timelines, citation bursts, keyword timelines, and keyword bursts. The co-occurrence function of CiteSpace identifies thematic clusters by visualizing frequently co-occurring items, while dual-map overlays reveal influential papers and cross-disciplinary connections by mapping citation patterns across disciplines. Specific parameter settings are detailed in the top left corner of the resultant images. Node size indicates the frequency of co-occurrence, while links denote co-occurrence relationships, with node and line colors corresponding to different years. Nodes identified by purple circles signify a high betweenness centrality (≥0.10), serving as crucial bridges between distinct networks (Chen, 2004). VOSviewer, another bibliometric tool, excels in generating knowledge maps illustrating clusters, overlays, or density colors. It was utilized for the co-occurrence analysis of co-cited authors and co-cited journals. In density maps, word and circle sizes, as well as the opacity of yellow, demonstrate a positive correlation with co-citation frequency. Excel software facilitated the analysis of annual publication trends and national outputs. Furthermore, the Bibliometrix R package 4.3.0 was utilized to create Three-Fields Plots. Additionally, we employed the online bibliometric analysis platform (https://bibliometric.com) to investigate collaboration networks among countries and publication output.

## 3 Results

### 3.1 Annual growth trend

Understanding the trend of development can be facilitated by statistically analyzing the number of publications each year. Following the predefined search strategy, a total of 623 relevant papers were retrieved from the WoSCC database, among which 606 eligible articles were ultimately included (Figure 1A; Supplementary Material 1). As depicted in Figure 1B, the number of articles related to ferroptosis in lung cancer has shown a steady increase over the past decade, rising from 1 article in 2015 to 211 articles in 2023, with further growth anticipated in 2024. From 2015 to 2020, there was a scarcity of relevant papers, but they exhibited a steady upward trend. However, post-2020, there has been a notable surge in the quantity of related publications. It is noteworthy that in 2023, the number of papers published reached 211, marking a 6.4-fold increase compared to 2020. This suggests significant advancements in the field or its related areas.

### 3.2 Distribution of countries/regions and institutions

**FIGURE 2:**
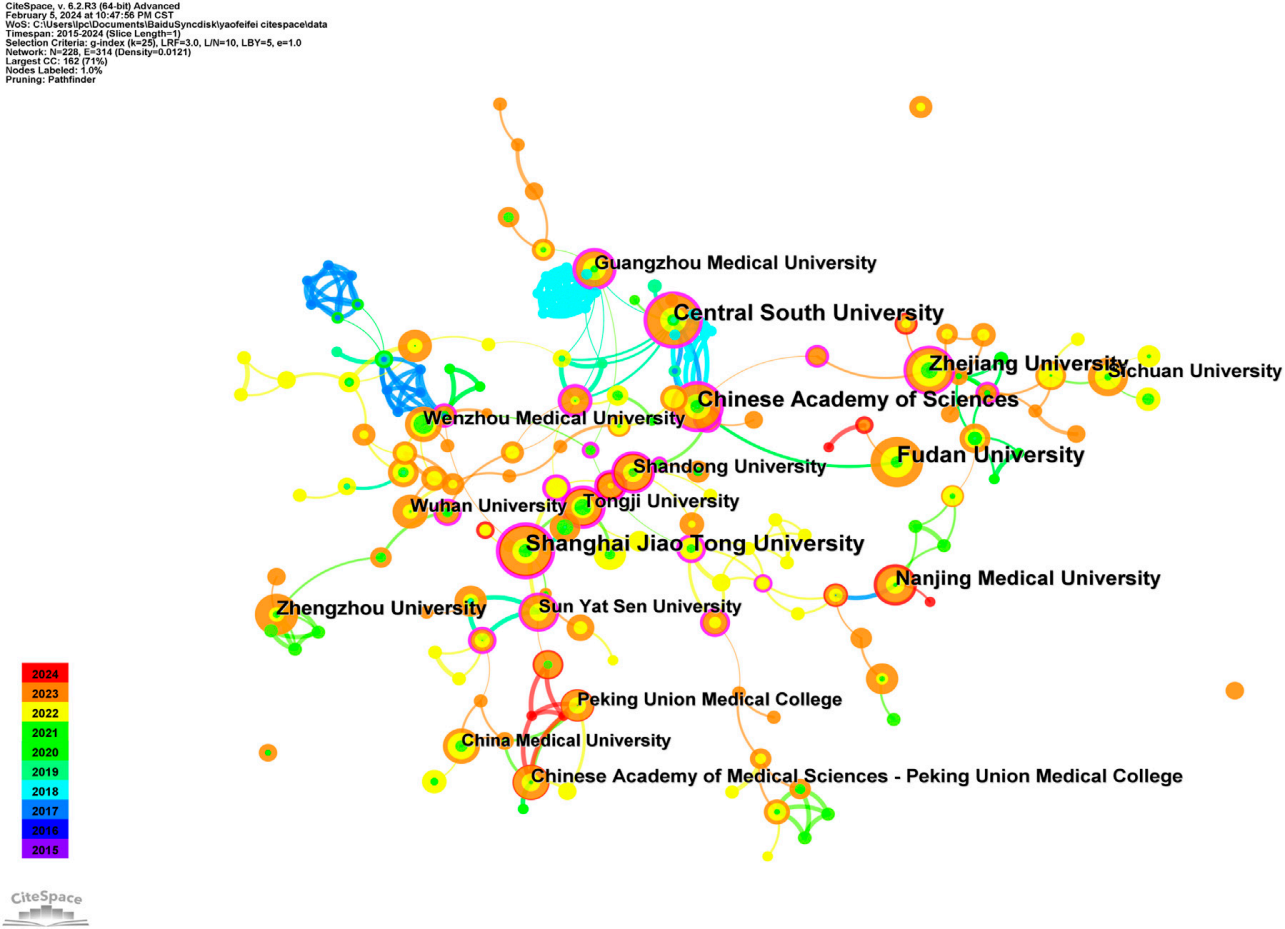
The co-occurrence map of institutions in the field of ferroptosis in lung cancer.

A collective of 606 papers stemmed from 42 distinct countries/regions and involved 758 institutions. Table 1 furnishes comprehensive details regarding the leading countries/regions and institutions associated with necroptosis. The highest number of publications came from China (n = 503), followed by the United States (n = 62), Germany (n = 22), Japan (n = 22), and Italy (n = 12) (see Figures 1C,D, 2). However, China’s centrality index was 0.16, significantly lower than that of the United States (0.56), indicating that despite China contributing to over 80% of the publications, its research impact is much lower than that of the United States, which remains a leading contributor in this field. As illustrated in Figure 2, Central South University (n = 28, centrality = 0.28), Shanghai Jiao Tong University (n = 28, centrality = 0.28), Fudan University (n = 27, centrality = 0.02), Chinese Academy of Sciences (n = 23, centrality = 0.36), and Zhejiang University (n = 22, centrality = 0.18) emerged as the top five productive institutions, all from China. Except for Fudan University, these institutions also exhibited relatively high centrality, indicating their increasing significance in the research landscape. Additionally, analysis of inter-institutional connections revealed active collaborations, predominantly initiated after 2020, suggesting that the field of ferroptosis in lung cancer is relatively young, with potential for increased collaborative achievements among global institutions in the future.

**TABLE 1:** Top 10 countries/regions and institutions related to ferroptosis in lung cancer.

No	Countries/Regions	Centrality	Count	Institution	Centrality	Count
1	Peoples R China	0.16	503	Central South University	0.28	28
2	United States	0.56	62	Shanghai Jiao Tong University	0.28	28
3	Germany	0.27	22	Fudan University	0.02	27
4	Japan	0.24	22	Chinese Academy of Sciences	0.36	23
5	Italy	0.16	12	Zhejiang University	0.18	22
6	South Korea	0.08	9	Nanjing Medical University	0.06	19
7	India	0.08	9	Zhengzhou University	0.06	17
8	Spain	0.57	6	Guangzhou Medical University	0.37	15
9	Iran	0	5	Tongji University	0.29	15
10	Belgium	0.15	4	Chinese Academy of Medical Sciences - Peking Union Medical College	0.37	15

### 3.3 Authors and co-cited authors

Table 2 illustrates the top 10 prolific researchers who have contributed to the exploration of ferroptosis in lung cancer, encompassing a total of 4033 authors. Lifang Ma from Shanghai Chest Hospital emerged as the most prolific author, followed by Xiao Zhang from Shanghai Jiao Tong University and Shuang Liu from Central South University. Ma’s pivotal contributions to lung adenocarcinoma (LUAD) research include examining N6-methyladenosine (m6A) RNA methylation’s role in ferroptosis and antioxidant pathways. They identified YTHDC2 as a LUAD tumorigenesis inhibitor and discovered connections between YTHDC2, SLC7A11, and SLC3A2, offering potential treatment strategies (Ma et al., 2021a; Ma et al., 2021b). Additionally, they explored other regulators such as CREB and IGF2BP3 in controlling ferroptosis sensitivity (Wang et al., 2021a; Xu et al., 2022). The scholar also uncovered a new regulatory axis involving YAP, TFCP2, and FTL affecting LUAD ferroptosis sensitivity and demonstrated the potential of 2D vermiculite nanosheets for effective LUAD treatment (Wang et al., 2021b; Ma et al., 2022). Their work significantly advances our understanding of LUAD pathogenesis and therapeutic interventions. Zhang’s research reveals crucial insights into LUAD, including YTHDC2’s inhibitory role, the significance of targeting SLC3A2 for ferroptosis therapy, and CREB’s regulation of GPX4 transcription. Additional findings involve DKK1, IGF2BP3, YAP-mediated transcriptional repression, and RB1CC1-mediated ferroptosis sensitization (Ma et al., 2021a; Ma et al., 2021b; Wang et al., 2021a; Wu et al., 2022a; Xu et al., 2022; Wang et al., 2021b; Xue et al., 2022). Liu Shuang and colleagues investigated lncRNA-microRNA interactions, chromatin modifiers, and ferroptosis in lung cancer, as well as the oncogenic role of GINS4, the tumor-suppressive function of GPR162 in radiotherapy, the impact of AhR on NSCLC, and the targeting of USP8 in HCC treatment. Taken together, these findings offer significant insights and promising therapeutic targets (Wang et al., 2019a; Yang et al., 2019; Chen et al., 2023a; Long et al., 2023; Peng et al., 2023; Tang et al., 2023).

**TABLE 2:** Top 10 authors and co-cited authors related to ferroptosis in lung cancer.

No	Author	Count	Centrality	Co-cited author	Count	Centrality
1	Ma, Lifang	7	0	Dixon SJ	345	0.24
2	Zhang, Xiao	7	0	Yang WS	236	0.16
3	Liu, Shuang	6	0	Stockwell BR	206	0.16
4	Zhan, Cheng	6	0	Chen X	158	0.03
5	Yu, Yongchun	6	0	Doll S	136	0.04
6	Wang, Jiayi	6	0	Jiang L	134	0.21
7	Tao, Yongguang	6	0	Angeli JPF	121	0.02
8	Xiao, Desheng	6	0	Hassannia B	121	0.13
9	Zhang, Jing	5	0	Wang WM	114	0.11
10	Qiu, Shiyu	5	0	Gao MH	107	0.07

**FIGURE 3:**
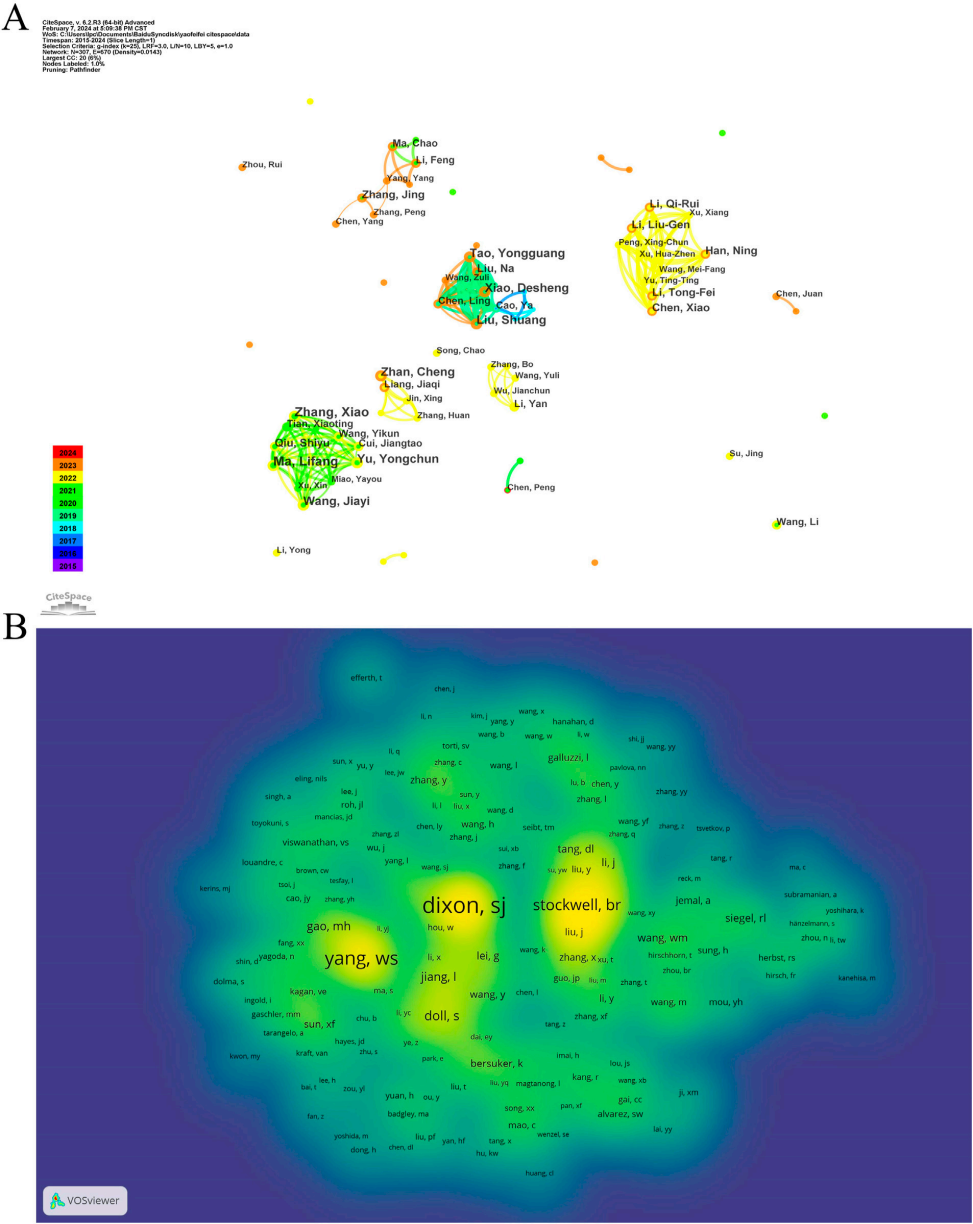
The co-occurrence authors’ **(A)** and co-cited authors’ **(B)** map of ferroptosis in lung cancer.

After a decade of development, scholars have gradually formed collaborative groups represented by six key researchers, including Lifang Ma, Liu Shuang, Zhan Cheng, Zhang Jing, Li Yan, and Chen Xiao (see Figure 3A). However, further analysis reveals a lack of effective communication and collaboration among these groups, which hinders disciplinary advancement. Strengthened cooperation among these groups is essential for future progress. In the domain of ferroptosis in lung cancer, a co-citation relationship arises when two or more authors are cited together in one or multiple papers. Out of 21,220 co-cited authors, 13 individuals have garnered over 100 co-citations each. Figure 3B presents a density plot that clearly illustrates high-frequency co-cited authors, with warmer colors indicating more citations. As shown in the table and figure, DIXON SJ, YANG WS, and STOCKWELL BR are the most frequently co-cited authors. DIXON SJ has significantly contributed to understanding ferroptosis regulation through lipid metabolism, emphasizing the role of lipid peroxidation and associated metabolic enzymes in this form of cell death (Pope and Dixon, 2023). YANG WS has provided comprehensive insights into the molecular ecosystem governing ferroptosis, including its heterogeneity and therapeutic potential across diseases (Dai et al., 2024). STOCKWELL BR, who coined the term “ferroptosis,” has extensively studied its mechanisms, physiological roles, and applications, laying the groundwork for therapeutic strategies targeting ferroptosis (Stockwell, 2022).

### 3.4 Journals and co-cited academic journals

**FIGURE 4:**
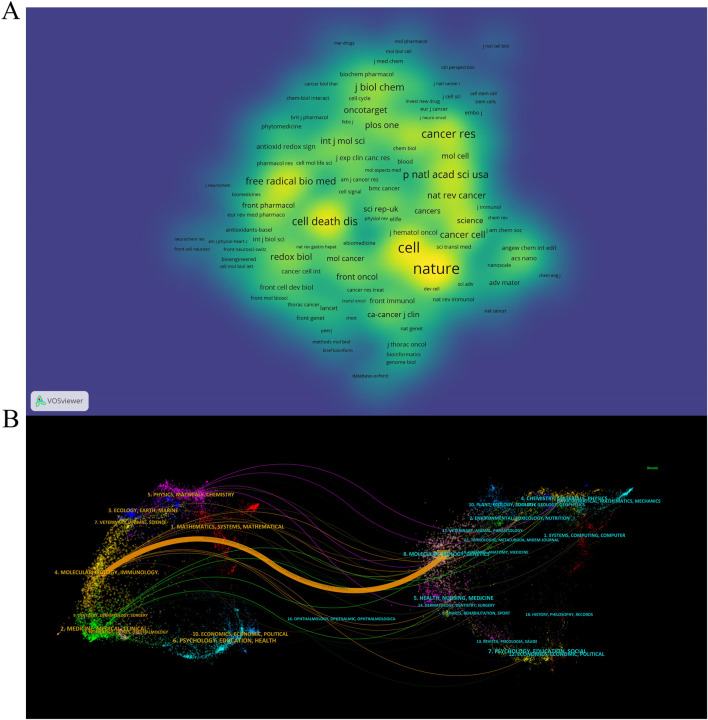
The density map of journals **(A)** and the dual-map overlay of journals **(B)** in the field of ferroptosis in lung cancer.

Academic journals play a critical role in disseminating scientific research findings, with a total of 262 academic journals publishing articles on ferroptosis in lung cancer. The top 5 journals collectively published 90 articles, constituting 14.9% of the total publications (refer to Table 3). Among these top 5 journals, three are categorized as JCR Q1 journals, predominantly focusing on cell biology and cell death research. Regarding publication volume, the most influential journal in this domain is “Frontiers in Pharmacology,” followed by “Frontiers in Oncology” and “Frontiers in Genetics.” As depicted in Figure 4A and detailed in Table 3, among the 2854 co-cited sources, 12 journals have accrued over 500 citations each, with “Nature” (n = 1,523), “Cell” (n = 1,331), and “Cancer Research” (n = 799) being the most frequently cited. Figure 4B illustrates the thematic distribution of journal relationships via a dual-map overlay. On the map, the left side represents the citing journals, while the right side indicates the cited journals. This visual representation elucidates a prominent citation pathway from Molecular/Biology/Immunology journals to Molecular/Biology/Genetic journals (z = 5.767, f = 12,052).

**TABLE 3:** Top 5 journals and co-cited journals related to ferroptosis in lung cancer.

No	Journal	Citation	JCR	Total link strength	Cited journal	Citation	JCR	Total link strength
1	Frontiers in Pharmacology	22	Q1	58	Nature	1523	Q1	151,950
2	Frontiers in Oncology	21	Q2	90	Cell	1331	Q1	120,902
3	Frontiers in Genetics	17	Q2	49	Cancer Research	799	Q1	97,792
4	Frontiers in Cell and Developmental Biology	16	Q1	60	Cell Death & Disease	782	Q1	94,833
5	Cell Death & Disease	14	Q1	52	Cell death and differentiation	626	Q1	70,693

### 3.5 Co-cited references and reference burst

Cited documents represent a repository of knowledge within a specific field, being cited by one or more publications simultaneously (Wang et al., 2022a). The analysis identifies 16 documents cited more than 50 times, including three research articles and seven review papers among the top 10, as shown in Table 4. The most cited document, published in Cell in 2017 by Brent R. Stockwell, reviews the iron-dependent regulated cell death process known as ferroptosis. The study explores ferroptosis’s role in various biological contexts, its association with diverse biological processes, and its links to a wide array of human diseases and heat stress in plants. Additionally, it discusses ferroptosis’s potential as a cancer therapy and provides guidelines and tools for further study (Stockwell et al., 2017). The second-ranked paper, authored by Weimin Wang et al. and published in Nature in 2019, investigates the function of CD8^+^ T cells and interferon gamma (IFNγ) in enhancing ferroptosis in tumor cells, thereby amplifying the effectiveness of cancer immunotherapy (Wang et al., 2019b). The paper demonstrates that IFNγ obstructs cystine uptake in tumor cells, triggering lipid peroxidation and subsequent ferroptosis. Augmenting this process through cystine deprivation and checkpoint blockade can enhance T-cell mediated anti-tumor immunity. Thus, promoting tumor ferroptosis could serve as a potential therapeutic strategy against tumor proliferation. These findings represent a significant contribution to the ongoing scientific discourse surrounding the intricate mechanisms driving ferroptotic processes in lung cancer. This iterative revelation will serve as a substantial stepping stone in the broader pursuit of curative and preventive measures against this lethal disease.

**TABLE 4:** Top 10 co-cited references related to ferroptosis in lung cancer.

Title	First author	Journals	Citations	Type	Year
Ferroptosis: A Regulated Cell Death Nexus Linking Metabolism, Redox Biology, and Disease	Brent R Stockwell	Cell	132	Review	2017
CD8^+^ T cells regulate tumour ferroptosis during cancer immunotherapy	Weimin Wang	Nature	113	Article	2019
Targeting Ferroptosis to Iron Out Cancer	Behrouz Hassannia	Cancer Cell	106	Review	2019
The CoQ oxidoreductase FSP1 acts parallel to GPX4 to inhibit ferroptosis	Kirill Bersuker	Nature	102	Article	2019
FSP1 is a glutathione-independent ferroptosis suppressor	Sebastian Doll	Nature	97	Article	2019
Broadening horizons: the role of ferroptosis in cancer	Xin Chen	Nat Rev Clin Oncol	90	Review	2021
Ferroptosis: mechanisms, biology and role in disease	Xuejun Jiang	Nat Rev Mol Cell Biol	68	Review	2021
Ferroptosis: past, present and future	Jie Li	Cell Death Dis	66	Review	2020
Ferroptosis, a new form of cell death: opportunities and challenges in cancer	Yanhua Mou	J Hematol Oncol	62	Review	2019
Ferroptosis at the crossroads of cancer-acquired drug resistance and immune evasion	José Pedro Friedmann Angeli	Nat Rev Cancer	60	Review	2019

**FIGURE 5:**
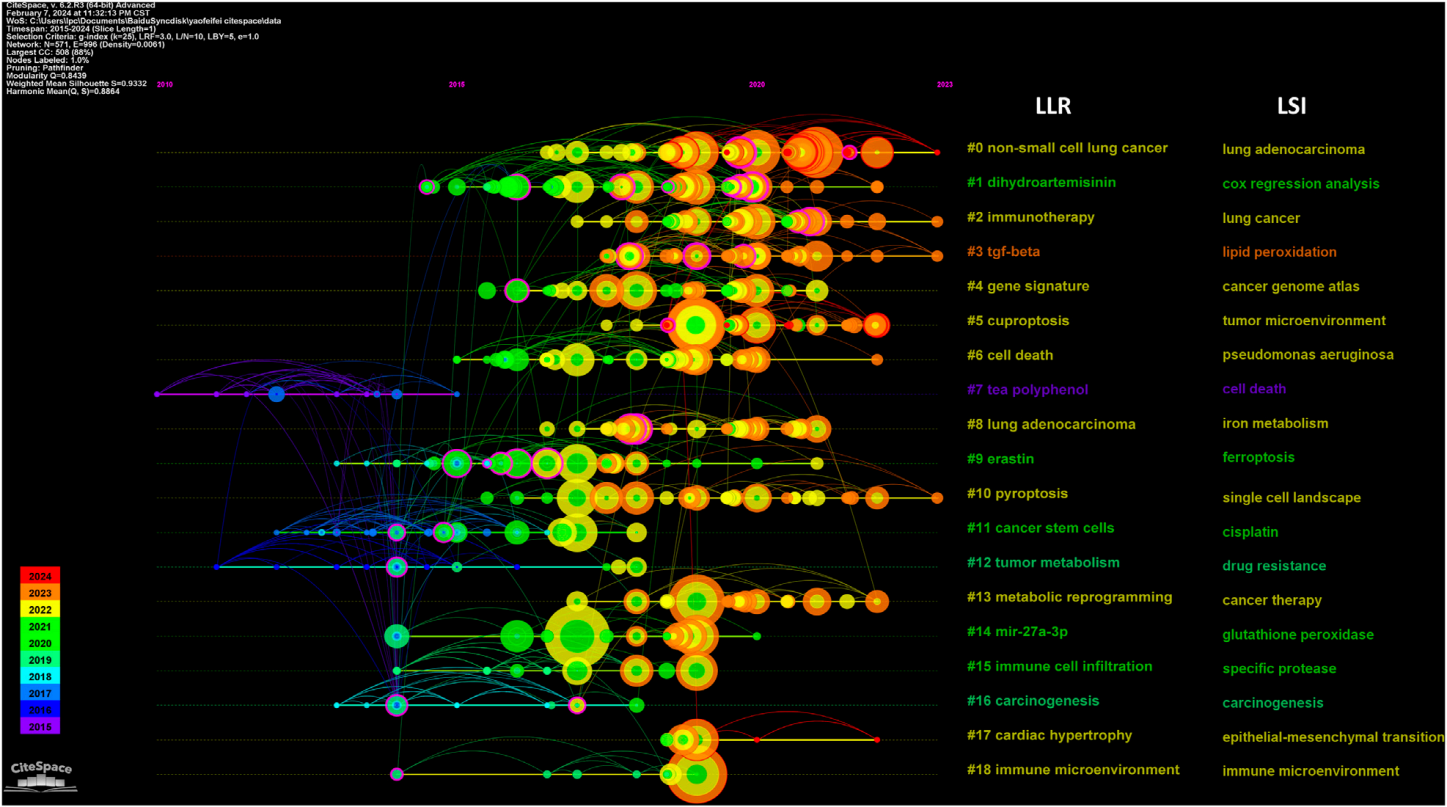
Timeline view of co-cited references related to ferroptosis in lung cancer.

**FIGURE 6:**
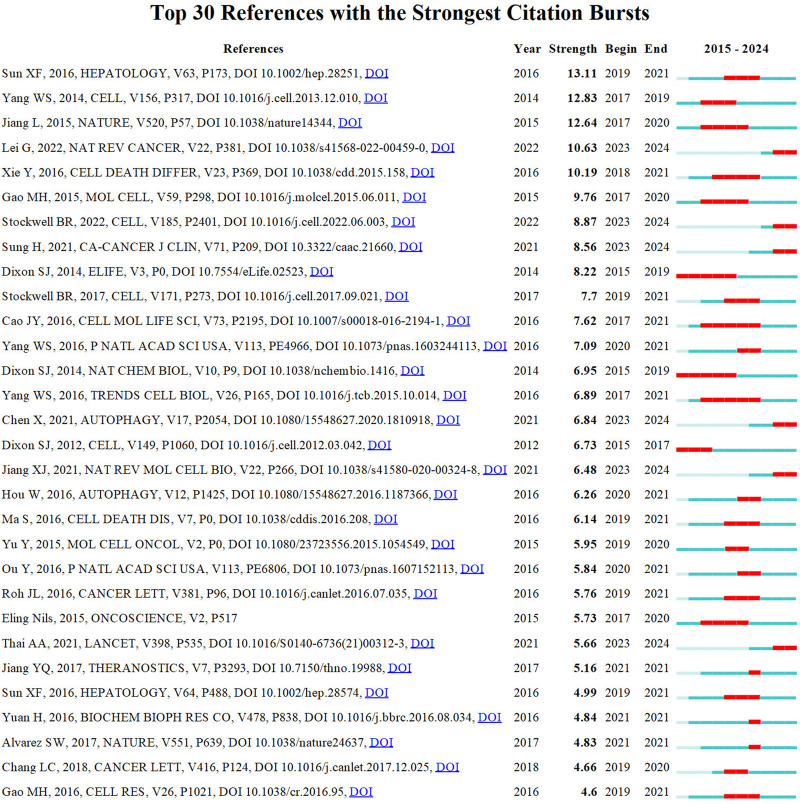
Top 30 references with the strongest citation bursts involved in ferroptosis in lung cancer.

The reference timeline view visualizes evolving research hotspots over time, with each cluster’s most frequent item marked as the cluster label. As depicted in Figure 5, clusters #7 (tea polyphenol/cell death), #9 (erastin/ferroptosis), #11 (cancer stem cells/cisplatin), #12 (tumor metabolism/drug resistance), and #16 (carcinogenesis/carcinogenesis) emerged earlier, while clusters 0 (non-small cell lung cancer/lung adenocarcinoma), 2 (immunotherapy/lung cancer), 3 (TGF-beta/lipid peroxidation), and 10 (pyroptosis/single cell landscape) are still ongoing and considered frontiers. Citation bursts refer to documents experiencing significantly higher citation rates within specific periods. Figure 6 displays the top 30 references exhibiting the strongest citation bursts. Based on the current understanding, future research on lung cancer ferroptosis should focus on understanding the oxidative stress-antioxidant defense mechanism relationship, identifying specific ferroptosis regulators, exploring ferroptosis’s complementarity with existing treatments, and developing new therapies to address therapy-resistant tumors. Due to visualization limitations in CiteSpace, not all information is displayed in the figures. Therefore, Supplementary Material 2 provides more detailed data.

### 3.6 Keyword analysis of trending research topic

**FIGURE 7:**
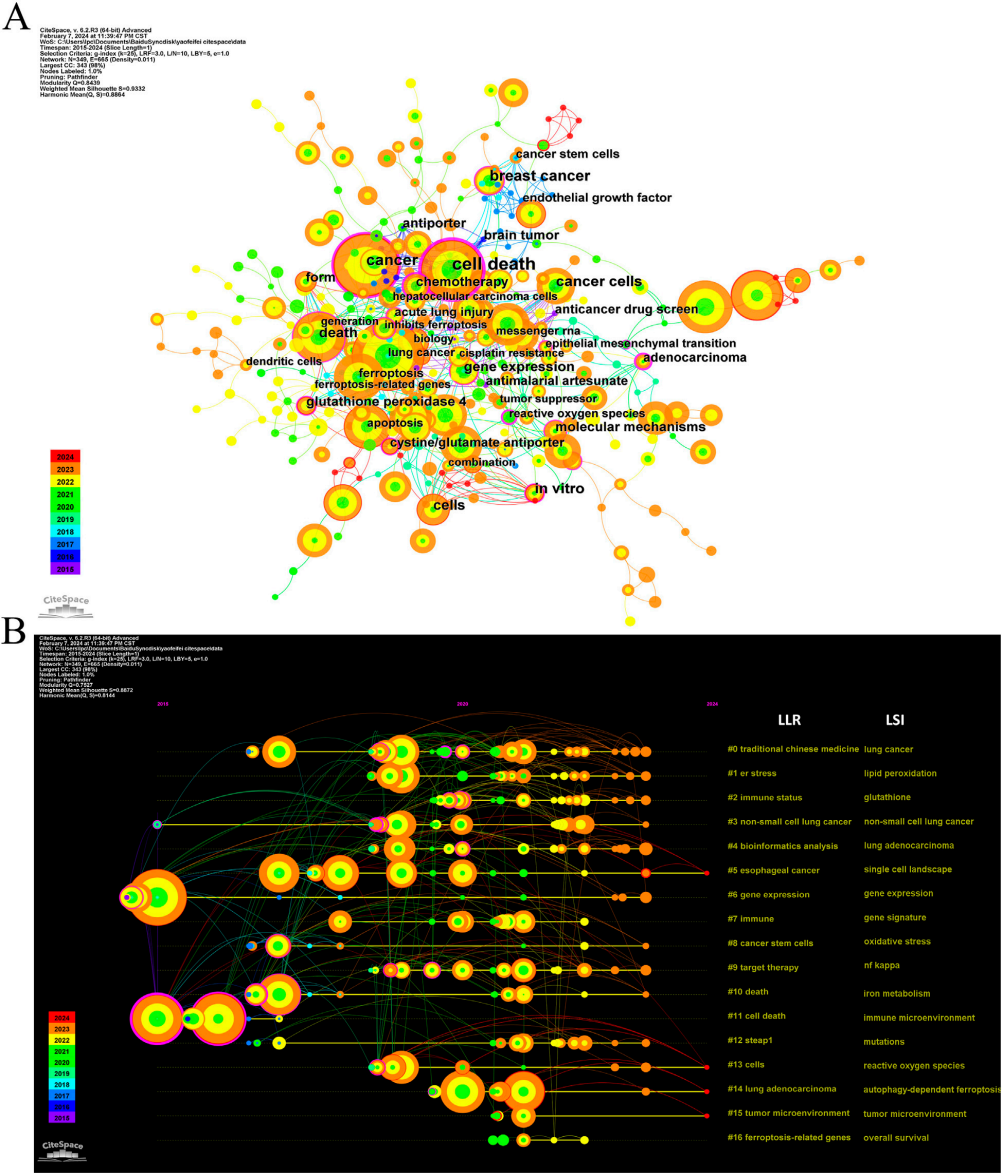
The co-occurrence network **(A)** and timeline view **(B)** of keywords related to ferroptosis in lung cancer.

**FIGURE 8:**
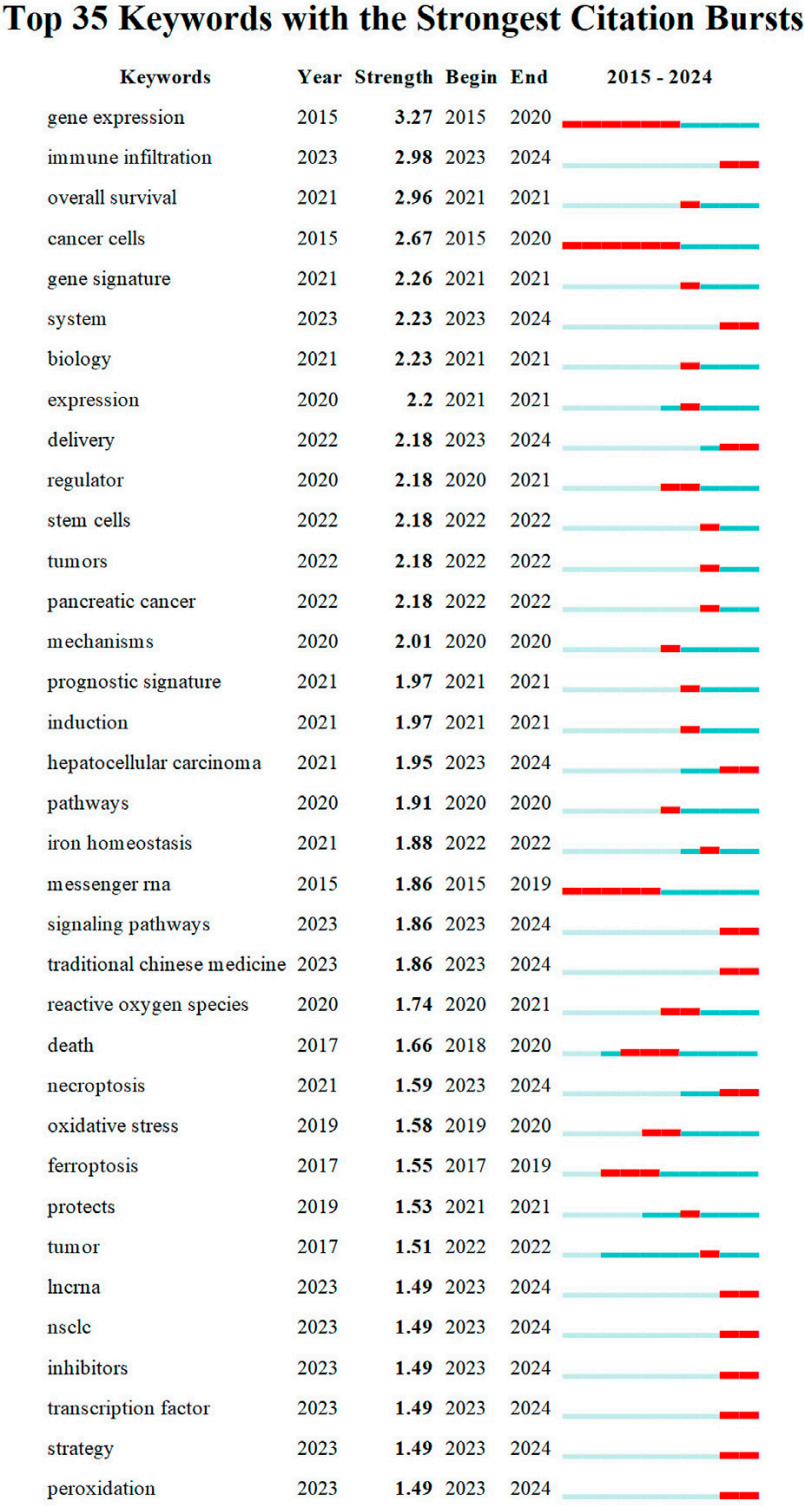
Top 35 keywords with the strongest citation bursts involved in ferroptosis in lung cancer.

**FIGURE 9:**
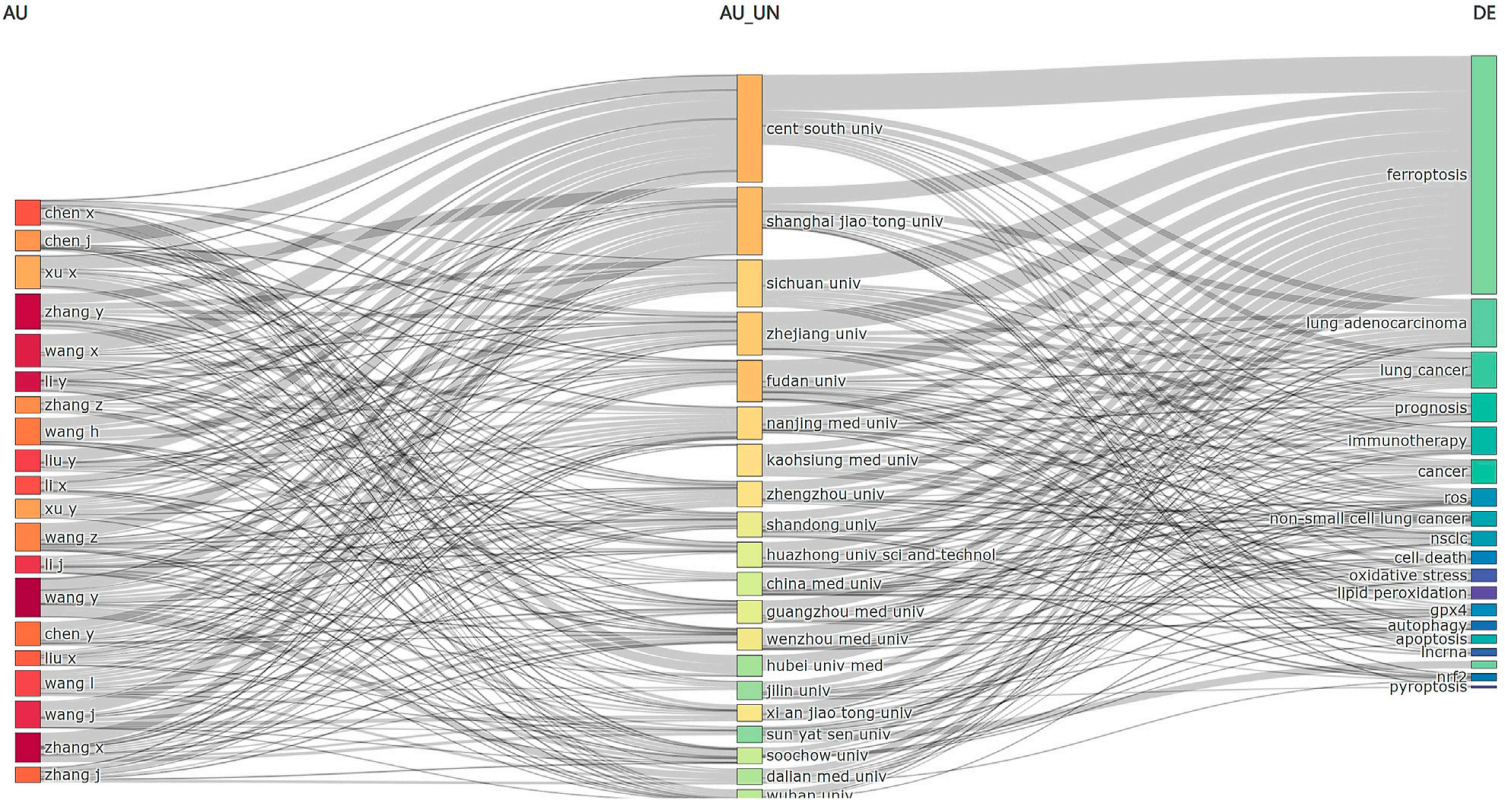
Three-field plot analysis related to ferroptosis in lung cancer. (left field: authors; middle field: affiliations; right field: keywords).

Keyword co-occurrence analysis offers valuable insights into research hotspots and directions within a specific field. A total of 2,366 keywords were extracted, with 69 keywords appearing 10 times or more, and 12 keywords appearing more than 50 times. As depicted in Table 5, “lung cancer” is the most common keyword (n = 149), followed by “cancer” (n = 122) and “cell death” (n = 112). Table 6 highlights the top 10 keywords related to ferroptosis, encompassing molecular and pathological processes. Notably, “apoptosis” (n = 67), “oxidative stress” (n = 58), “lipid peroxidation” (n = 37), “tumor microenvironments” (n = 24), “autophagy” (n = 24), “drug resistance” (n = 19), “nanoparticles” (n = 19), “glutathione” (n = 15), “nrf2” (n = 12), and “gpx4” (n = 13) represent the most studied molecules or pathological processes. Among these, “lipid peroxidation” serves as a hallmark of ferroptosis, closely linked to oxidative stress and cell death mechanisms. Recent studies emphasize its critical role in lung cancer progression through the amplification of oxidative damage in tumor cells (Jelic et al., 2021). Similarly, “tumor microenvironment” highlights the role of ferroptosis in modulating immune responses and therapeutic resistance within cancerous tissues, as supported by emerging evidence on its influence on stromal interactions and immune infiltration (Wang et al., 2022b), (Jiao et al., 2024). Additionally, “autophagy” reflects its dual regulatory role in ferroptosis initiation and progression, offering potential therapeutic targets. Figure 7A illustrates a keyword co-occurrence network in the field of ferroptosis in lung cancer, where colors denote the average publication year, node size reflects the frequency of keyword usage, and distances between nodes signify the strength of relationships. Figure 7B presents a timeline view showing the evolution of each cluster over time. The largest cluster, #0 (traditional Chinese medicine/lung cancer), is followed by #1 (ER stress/lipid peroxidation) and #2 (immune status/glutathione). Keyword bursts signify keywords that are frequently cited within a certain period. As demonstrated in Figure 8, “gene expression” has the strongest burst intensity (intensity = 3.27), followed by “immune infiltration” (intensity = 2.98) and “overall survival” (intensity = 2.96). It is noteworthy that as of 2024, keywords such as “immune infiltration,” “system,” and “delivery” continue to experience bursts, offering insights for scholars to anticipate future research directions. Figure 9 further elucidates the associations between authors, affiliations, and keywords in the research field of ferroptosis in lung cancer.

**TABLE 5:** Top 20 keywords related to ferroptosis in lung cancer.

Rank	Keywords	Counts	Rank	Keywords	Counts
1	Lung cancer	149	11	Resistance	55
2	Cancer	122	12	Activation	55
3	Cell death	112	13	Metabolism	53
4	Expression	86	14	Cancer cells	49
5	Death	82	15	Iron	46
6	Lung adenocarcinoma	79	16	Mechanisms	38
7	Ferroptosis	73	17	Proliferation	38
8	Apoptosis	67	18	Cells	38
9	Oxidative stress	58	19	Lipid peroxidation	37
10	Lung cancer	149	20	Non-small cell lung cancer	35

**TABLE 6:** Top 10 molecules or pathological process related to ferroptosis in lung cancer.

Rank	Molecules	Counts	Year	Rank	Molecules	Counts	Year
1	Apoptosis	67	2018	6	Drug resistance	19	2020
2	Oxidative stress	58	2019	7	Nanoparticles	19	2021
3	Lipid peroxidation	37	2019	8	Glutathione	15	2020
4	Tumor microenvironment	24	2021	9	nrf2	14	2021
5	Autophagy	24	2021	10	gpx4	13	2020

## 4 Discussion

### 4.1 General information and knowledge base

This study’s bibliometric analysis provides significant insights into the trends, key contributors, and research focal points concerning ferroptosis in lung cancer. Notably, there has been a consistent rise in publications over the past decade, with a notable spike observed in 2020. This surge can be attributed to several significant advancements: (1) In 2019, Sebastian Doll’s team discovered a novel mechanism involving FSP1-CoQ10-NAD(P)H, providing a new drug development target to inhibit ferroptosis in susceptible cancer cells (Doll et al., 2019). (2) In the same year, researchers from the Air Force Medical University of China and the Memorial Sloan Kettering Cancer Center in the United States found that cadherin mediates cell-cell interactions regulating ferroptosis (Wu et al., 2019). (3) Researchers from the University of Michigan and Cayman Chemical Company discovered that T cell-promoted tumor ferroptosis is an anti-tumor mechanism, proposing a potential therapeutic approach (Wang et al., 2019b). These findings have sparked enthusiasm among scientists, significantly advancing the development of ferroptosis in lung cancer. In examining countries/regions, both the volume of publications and betweenness centrality serve as pivotal indicators. China leads in publication quantity, indicating significant investment in cancer research, including ferroptosis. Nevertheless, despite China’s substantial publication count, its centrality falls below that of the United States. This discrepancy may stem from the United States’ extensive international collaborations and the broader global impact of its research in ferroptosis, which reinforce its dominant position in this field.

Prominent contributors in this field include Mali Fang, Xiao Zhang, and Shuang Liu, focusing on understanding ferroptosis’s molecular mechanisms and therapeutic implications in lung cancer. While research clusters have formed among these key researchers, enhancing communication and collaboration is essential for interdisciplinary progress. Scott J. Dixon, a leading scholar, has made significant contributions by elucidating ferroptosis mechanisms and developing molecular targeting tools (Dixon et al., 2012; Galluzzi et al., 2018; Pope and Dixon, 2023; Cao and Dixon, 2016; Dixon and Olzmann, 2024; Hendricks et al., 2023).

Journal and co-cited journal analysis reveal the dissemination and impact of research achievements. “Frontiers in Pharmacology” and “Frontiers in Oncology” play crucial roles in disseminating studies on lung cancer ferroptosis, while “Nature” and “Cell” are frequently cited, focusing on molecular biology, immunology, and genetics, consistent with the dual-map analysis.

Figure 6 illustrates the top 30 references with the strongest citation bursts, comprising six papers currently undergoing bursts. These findings provide valuable insights for anticipating future research directions. Future research should delve into the complex relationship between oxidative stress, antioxidant defense mechanisms, and their connection to lung cancer, potentially unveiling new treatment options (Chen et al., 2021; (Jiang et al., 2021). Investigating ferroptosis’s molecular mechanisms and physiological functions may lead to breakthroughs, and targeting specific regulators within metabolic frameworks holds promise for novel therapies (Stockwell, 2022).

Other mechanisms in lung cancer, such as immunity or immunotherapy, should also be explored. For example, a recent study demonstrated that rescheduling anti-VEGF/cytotoxics/anti-PD-1 combinations could effectively overcome immuno-resistance in lung cancer models (Sicard et al., 2024). Additionally, the diagnostic and prognostic aspects of lung cancer require attention. The genomic instability-derived predictive prognostic signature provides a reliable tool for predicting outcomes in non-small cell lung cancer patients (Li et al., 2023a). Exosome signaling represents another promising area; for instance, the RNA profile of immuno-magnetically enriched lung cancer-associated exosomes has highlighted disease-specific transcriptomes as potential biomarkers (Singh et al., 2023).

Integrating ferroptosis-inducing strategies with current treatments could revolutionize lung cancer management and improve patient outcomes (Lei et al., 2022; Thai et al., 2021). In conclusion, future research should deepen our understanding of ferroptosis, develop targeted therapies, and integrate new approaches with existing treatments to address therapy-resistant lung cancer effectively (Sung et al., 2021).

### 4.2 Future directions and research hotspots

Research hotspots and future research directions are widely discussed scientific topics, which are also among the questions this study seeks to address. Based on analyses from various perspectives such as authors, journals, literature, and keywords, we summarize the following potential research hotspots and future research directions:

#### 4.2.1 Mechanism of occurrence

Lung cancer tissues commonly exhibit higher levels of reactive oxygen species and lipid oxidation markers. Enhancing the synthesis of glutathione (GSH) or boosting the activity of the Xc- system or GPX4 can protect cells from various oxidative stresses, particularly cell death induced by thiol deficiency. These early studies underscore the close relationship between lung cancer and ferroptosis. Building on advancements in research over the past decade, we propose several potential research directions for elucidating the mechanisms of ferroptosis in lung cancer:

① Endoplasmic Reticulum (ER) Stress: Currently, extensive research focuses on the association between ferroptosis and oxidative stress in lung cancer (Panda et al., 2023). However, studies investigating the relationship between ferroptosis and endoplasmic reticulum (ER) stress in lung cancer remain relatively limited. ER stress is a protective mechanism cells employ to cope with external pressures; however, sustained or severe stress can lead to cell death (Borok et al., 2020). Recent studies have shed light on the intricate relationship between ferroptosis and ER stress in the context of lung cancer. Fu et al. developed inhalable biomineralized liposomes containing both dihydroartemisinin (DHA) and pH-responsive calcium phosphate (CaP) to trigger ferroptosis in lung cancer cells. The degradation of CaP shell triggered a Ca2+ burst-mediated intense ER stress, enhancing ROS accumulation and reinforcing ferroptosis. This Ca2+ burst-centered ER stress mechanism highlights the interplay between ferroptosis and ER stress pathways in lung cancer therapy (Fu et al., 2023). Similarly, Han et al. demonstrated that DHA treatment induced ferroptosis in lung cancer cells, concomitant with the stimulation of ER stress and DNA damage. Ferroptosis inhibitors attenuated ER stress and DNA damage induced by DHA, underscoring the crosstalk between ferroptosis and ER stress pathways in lung cancer immunogenicity (Han et al., 2023). Additionally, Li et al. found that melittin-induced ferroptosis in A549 lung cancer cells was accompanied by ER stress-mediated CHOP-dependent apoptosis (Li et al., 2022). These findings collectively emphasize the significance of the interplay between ferroptosis and ER stress pathways in regulating cell death mechanisms in lung cancer. Understanding the molecular intricacies of this relationship may offer new insights into developing targeted therapeutic interventions for lung cancer treatment.

② Tumor Microenvironment (TME): The tumor microenvironment (TME) plays a crucial role in various stages of cancer development. Most inflammatory cells and pro-inflammatory factors in the TME can promote tumor initiation and progression by inhibiting the ferroptosis pathway in tumor cells (Xian et al., 2020). Cancer-associated fibroblasts (CAFs), as the most important stromal cells in the TME, are involved in all stages of tumor development (Denton et al., 2018; Zhang et al., 2020). Additionally, angiogenesis is essential for the rapid growth of malignant tumors. Hypoxia, under specific circumstances, stimulates angiogenesis, a process regulated by the hypoxia-inducible factor (HIF) transcription factor family (Lugano et al., 2020). Studies have reported that the instability of HIF-1α in non-small cell lung cancer cells diminishes their vulnerability to ferroptosis (Jiang et al., 2017). Based on this evidence, we believe that the relationship between the tumor microenvironment (TME) and ferroptosis is key to shaping tumor behavior and therapeutic responses. In lung cancer, recent investigations have revealed distinct molecular subtypes influenced by ferroptosis-related genes, alongside varied TME cell infiltration patterns. Zhang et al. identified three lung cancer subtypes mediated by ferroptosis, exhibiting diverse prognoses and immune characteristics. Notably, low ferroptosis scores correlated with immune activation and favorable outcomes, whereas high scores associated with immunosuppression and poorer prognoses (Zhan et al., 2021b). These findings resonate with Mao’s study on non-small cell lung cancer (NSCLC), where targeting cholesterol synthesis with statin induced ferroptosis and transformed the TME from immuno-cold to inflamed, enhancing the response to immune checkpoint blockade (ICB) therapy (Mao et al., 2022). Additionally, Hsieh’s novel NRF2 nano-modulator triggered ferroptosis in lung cancer cells, simultaneously fostering an immunostimulatory tumor microenvironment (TME). This underscores the potential of combined approaches to synergistically induce cancer cell demise and reshape the TME towards anti-tumor immune responses (Hsieh et al., 2021). However, research on the tumor microenvironment remains limited, and the related mechanisms have not been fully elucidated. Clinical applications are still distant. Therefore, future research directions may involve elucidating the intricate interplay between ferroptosis, TME modulation, and therapeutic responses, aiming to optimize treatment strategies and improve outcomes for patients with lung cancer. Additionally, exploring the broader applicability of ferroptosis modulation in various cancer types and assessing combinatorial approaches with immunotherapies could pave the way for more effective and personalized anticancer interventions.

③ Autophagy: Autophagy emerges as a critical regulator of ferroptosis in lung cancer, offering novel insights into therapeutic strategies (Gao et al., 2022). Bhatt et al. uncovered that in LKB1-deficient KRAS-driven NSCLC, autophagy upregulation promotes resistance to MEK inhibition by inhibiting ferroptosis, highlighting the potential of autophagy inhibition combined with MEK inhibition as a therapeutic avenue (Bhatt et al., 2023). Tang et al.'s study further elucidates this connection, demonstrating that curcumin induces ferroptosis in NSCLC through activating autophagy, thereby enhancing its anti-tumor effects (Tang et al., 2021). These discoveries highlight the complex interconnection between autophagy and ferroptosis concerning the advancement of lung cancer and the response to treatment. Future research may target new therapies to exploit the autophagy and ferroptosis relationship, enhancing treatment outcomes and understanding resistance mechanisms (Xia et al., 2019).

#### 4.2.2 Cancer treatment

Ferroptosis, identified as a regulated cell demise process dependent on iron-induced lipid peroxidation, has surfaced as a hopeful avenue in the treatment of lung cancer. Combined with the current literature, we have identified some ferroptosis targets that may guide future research directions (Song and Long, 2020).

① GPX4: Among the most classic targets of ferroptosis, GPX4 has a negative effect on ferroptosis in various cancer cells. In studies focusing on lung cancer, directing efforts towards GPX4 has demonstrated the capability to trigger ferroptosis in lung adenocarcinoma cells. This intervention effectively overcomes their resistance to epidermal growth factor receptor tyrosine kinase inhibitors (EGFR-TKIs), highlighting the potential of GPX4 as a viable target against EGFR-TKIs resistance in LUAD (Zhang et al., 2022b). Furthermore, focusing on GPX4 has been shown to enhance lapatinib-induced ferroptosis in drug-resistant non-small cell lung cancer cells (Ni et al., 2021). Moreover, GPX4 holds clinical significance in reducing lung cancer radiotherapy resistance (Pan et al., 2019; Liu et al., 2023b). Recent reviews emphasize the role of GPX4 as a critical modulator of ferroptosis and its potential in overcoming drug resistance in cancer treatments (Lou et al., 2021).

② SLC7A11: Elevated in multiple human cancers, SLC7A11 facilitates tumor survival (Koppula et al., 2021; Chen et al., 2023b). Wu et al. conducted RNAseq and miRNAseq data analysis from The Cancer Genome Atlas (TCGA) and found significant dysregulation of lncRNA, miRNA, and mRNA in lung adenocarcinoma. They further constructed a lung adenocarcinoma-specific ceRNA network, identifying key interactions influencing tumor-related molecular functions and pathways. SLC7A11 was discovered among the 16 mRNAs associated with overall survival in lung adenocarcinoma patients (Wu et al., 2020). Further mechanistic exploration revealed that lung cancer stem-like cells (CSLCs) resist ferroptosis by upregulating the cystine transporter SLC7A11, activated by the stem cell transcription factor SOX2. Mutation of the SOX2 binding site in the SLC7A11 promoter enhances cancer cell sensitivity to ferroptosis, proposing SLC7A11 as a potential therapeutic target for lung cancer treatment (Wang et al., 2021c). Recent literature highlights the importance of SLC7A11 in modulating ferroptosis resistance in cancer and its emerging potential as a target for combination therapies (Li et al., 2023b).

③ P53: The cell signaling pathway mediated by P53, acting as a tumor suppressor, significantly regulates normal cellular activities. p53 mediates the downregulation of H2Bub1 levels by facilitating the nuclear translocation of the deubiquitinase USP7. Consequently, this reduces H2Bub1 occupancy on the SLC7A11 gene regulatory region, leading to the repression of SLC7A11 expression during ferroptosis induction. These discoveries highlight a novel aspect of p53 in chromatin regulation and establish a connection between p53 and ferroptosis through an H2Bub1-mediated epigenetic pathway (Wang et al., 2019c). Furthermore, due to the challenge of drug resistance, the clinical application of ferroptosis-inducing agents faces obstacles. Emerging nanoplatform-based strategies offer a potential solution. Specifically, a metallo-organic supramolecular construct (Nano-PMI@CeO2) is designed to restore P53 function and sensitize ferroptosis synergistically. Within this configuration, CeO2 nanoparticles serve as the core, inducing radical generation, while encapsulated within the shell is a p53-activator peptide (PMI) bonded to a gold precursor polymer. Nano-PMI@CeO2 effectively reactivates the P53 pathway, downregulating GPX4 expression, and inhibiting tumor progression in lung cancer models while ensuring biosafety. This approach presents a dual therapeutic strategy for inducing ferroptosis and activating P53, demonstrating potential in sensitizing ferroptosis through P53 activation (Wang et al., 2022c). Recent reviews have suggested that combining p53 reactivation with ferroptosis induction could offer promising therapeutic outcomes, particularly in overcoming drug resistance (Hassannia et al., 2019).

④ FSP1: Expression of FSP1 is positively correlated with lung cancer cell resistance to ferroptosis (Bersuker et al., 2019). A recent investigation unveiled the CoQ-FSP1 axis as a pivotal mechanism driving both ferroptosis and resistance to radiation therapy in lung cancers lacking KEAP1 activity. FSP1, regulated by NRF2, mediates this resistance mechanism. Blocking the CoQ-FSP1 axis renders KEAP1-mutant lung cancer cells more vulnerable to radiation therapy by triggering ferroptosis, indicating FSP1 as a promising therapeutic target for combating treatment resistance in these malignancies (Koppula et al., 2022).

Recent studies underscore the importance of ferroptosis modulation in overcoming treatment resistance in lung cancer, particularly when integrated with existing therapies. For example, ferroptosis induction has been shown to enhance the efficacy of chemotherapy, radiotherapy, and targeted therapies by sensitizing resistant cancer cells (Zhang et al., 2022b; Ni et al., 2021, (Pan et al., 2019). Targets such as Nrf2 (Lou et al., 2021; Takahashi et al., 2020; Wang et al., 2023), LSH (Ho and Crabtree, 2010; Mao et al., 2018; Jiang et al., 2017; Wang et al., 2019a), STYK1/NOK (Ma et al., 2019; Lai et al., 2019), and ACSL4 (Doll et al., 2017; Kuwata and Hara, 2019; Hou et al., 2022) also hold significant promise in the field of ferroptosis in lung cancer.

#### 4.2.3 Traditional Chinese medicine

Traditional Chinese medicine (TCM) boasts a rich history and is widely utilized in cancer treatment. Diverging from modern medicine, TCM adopts a holistic approach, treating patients as integrated systems, and employs herbal formulations to tackle multifaceted diseases like cancer (Wu et al., 2022b; Wen et al., 2023). Recent studies have uncovered the potential therapeutic effects of certain TCM formulations on lung cancer by promoting ferroptosis. For instance, Hedyotis diffusa injection (HDI) has been found to induce ferroptosis in lung adenocarcinoma cells by increasing ROS release through the Bax/Bcl2/VDAC2/3 axis, independently of GPX4 and the PUFA-PLS pathway (Huang et al., 2022).

However, traditional Chinese herbal medicine typically comprises a complex mixture of multiple active ingredients, rendering its pharmacological effects and mechanisms intricate and diverse, challenging to elucidate and verify. Furthermore, compared to single-molecule drugs, the composition and production process of Chinese herbal medicine are influenced by various factors such as geography, season, and growing conditions, resulting in batch variations and a lack of standardized quality and production processes (Tu, 2016; Wang et al., 2018). Acknowledging these constraints, China has actively advocated for the modernization of TCM in recent years. Research on effective monomeric components of TCM stands as a pivotal scientific issue in TCM development and application, serving as a vital source for novel drug development. Leveraging computer-assisted techniques and modern analytical methods to explore the molecular targets and efficacy mechanisms of TCM monomers will furnish critical scientific foundations for comprehending the scientific essence of TCM and its global integration (Zhang et al., 2023).

Luteolin, a natural flavonoid extracted from various fruits, vegetables, and medicinal herbs, showcases significant anti-tumor, anti-inflammatory, anti-bacterial, and neuroprotective properties. Zheng et al. discovered that luteolin and erastin synergistically induce ferroptosis in colon cancer cells by downregulating GPX4 via HIC1. This combination diminishes cell viability, heightens lipid peroxides, and reduces glutathione levels *in vitro*. *In vivo* experiments demonstrate its ability to curb xenograft growth, hinting at a promising therapeutic avenue for colon cancer treatment (Zheng et al., 2023). Nonetheless, research on the effects of TCM monomers on ferroptosis in lung cancer remains scarce, representing a relatively unexplored research domain. Furthermore, the mechanisms of TCM in ferroptosis require further nuanced exploration, especially considering the emerging interest in integrating TCM with conventional treatments. For example, Liu et al. suggested that traditional toxin medicines, commonly used in TCM, may exert anti-cancer effects through ion channel regulation (Hengrui, 2022). This perspective highlights the importance of identifying effective components and pharmacological targets to enhance TCM’s clinical application. Similarly, a follow-up commentary emphasized this by providing specific examples of TCM toxin medicines in cancer treatment (Hengrui, 2022). How to integrate TCM with ferroptosis in lung cancer and surmount the deficiencies in lung cancer treatment poses a question that demands the attention of many TCM formulation researchers.

In addition to the previously discussed research directions, further exploration into the identification of biomarkers capable of predicting lung cancer patients’ responses to ferroptosis-induced therapy is crucial. These biomarkers could be instrumental in determining, prior to treatment, which patients are most likely to benefit from ferroptosis-based therapies, thus ensuring a more personalized and effective approach (Conche et al., 2023; Chen et al., 2023c). Personalized treatment strategies, driven by biomarkers, would not only minimize unnecessary treatments but also maximize therapeutic outcomes by selecting the most suitable therapies for each individual. Moreover, another promising area of research lies in the development of innovative drug delivery systems, such as aerosol drug delivery (Chandel et al., 2019; Reychler and Michotte, 2019). This approach could significantly improve the targeting and accumulation of ferroptosis-inducing agents directly within lung tumor lesions, enhancing their therapeutic efficacy. The ability to concentrate therapeutic agents more precisely in tumor areas while minimizing systemic side effects could greatly optimize the potential of ferroptosis-based treatments in lung cancer. Together, these avenues—biomarker identification and advanced drug delivery systems—represent significant frontiers that could shape the future of lung cancer therapy, ensuring more precise, efficient, and patient-tailored interventions.

### 4.3 Limitations

While bibliometric analysis yields valuable insights, it is not without its limitations. Relying solely on specific publication databases, such as Web of Science or Scopus, can introduce selection bias due to the varying coverage and indexing criteria of these databases. Additionally, the exclusion of non-English publications limits the global representation of research findings, particularly from regions where non-English works predominate. Moreover, interpreting bibliometric data necessitates careful consideration of environmental factors and qualitative insights. Nevertheless, despite these limitations, we can reasonably infer that our study provides a broad overview of the landscape and emerging trends in this field.

## 5 Conclusion

In summary, this analysis highlights key trends, major contributors, and emerging research frontiers in the field of ferroptosis in lung cancer. Over the past decade, the United States has maintained a leading role in advancing ferroptosis research in this context, with Scott J. Dixon emerging as the most influential author in the field. Current research efforts are primarily focused on understanding the mechanisms that drive the initiation of ferroptosis in lung cancer and exploring potential therapeutic strategies to target this pathway. Additionally, while traditional Chinese medicine shows promise in inducing ferroptosis in lung cancer cells, further studies are needed to clarify its underlying mechanisms and address challenges related to its complex composition and production processes.

Despite the promising theoretical advancements, translating bibliometric findings into practical cancer therapies remains challenging. The complexity of ferroptosis regulation, variability in patient responses, and difficulties in developing targeted therapies complicate the translation of research into clinical practice. These obstacles highlight a significant gap between scientific discoveries and their application in the clinic, underscoring the urgent need for more translational research.

To bridge this gap, further research is required to identify biomarkers capable of predicting lung cancer patients’ responses to ferroptosis-based therapies. Such biomarkers would be instrumental in selecting patients who are most likely to benefit from ferroptosis-induced treatments, as individual responses to therapy can vary considerably. Biomarker detection could facilitate the determination of the most effective therapeutic strategy for each patient, reducing unnecessary treatments and enhancing overall efficacy. This personalized approach would ensure that patients receive interventions tailored to their unique molecular profiles, optimizing therapeutic outcomes. Identifying reliable biomarkers for ferroptosis susceptibility is therefore critical for advancing clinical applications and improving lung cancer treatment.

Overall, this study provides a comprehensive review of the current state of ferroptosis research in lung cancer, offering valuable insights into its potential for future therapeutic strategies.

## Data Availability

The original contributions presented in the study are included in the article/Supplementary Material, further inquiries can be directed to the corresponding author.

## References

[B1] Bersuker K. Hendricks J. M. Li Z. Magtanong L. Ford B. Tang P. H. (2019). The CoQ oxidoreductase FSP1 acts parallel to GPX4 to inhibit ferroptosis. Nature 575 (7784), 688–692. 10.1038/s41586-019-1705-2 31634900 PMC6883167

[B2] Bhatt V. Lan T. Wang W. Kong J. Lopes E. C. Wang J. (2023). Inhibition of autophagy and MEK promotes ferroptosis in Lkb1-deficient Kras-driven lung tumors. Cell Death Dis. 14 (1), 61. 10.1038/s41419-023-05592-8 36702816 PMC9879981

[B3] Borok Z. Horie M. Flodby P. Wang H. Liu Y. Ganesh S. (2020). Grp78 loss in epithelial progenitors reveals an age-linked role for endoplasmic reticulum stress in pulmonary fibrosis. Am. J. Respir. Crit. Care Med. 201 (2), 198–211. 10.1164/rccm.201902-0451OC 31738079 PMC6961744

[B4] Cao J. Y. Dixon S. J. (2016). Mechanisms of ferroptosis. Cell Mol. Life Sci. 73 (11-12), 2195–2209. 10.1007/s00018-016-2194-1 27048822 PMC4887533

[B5] Chai M. Li X. Zhang Y. Tang Y. Shu P. Lin J. (2021). A nomogram integrating ferroptosis- and immune-related biomarkers for prediction of overall survival in lung adenocarcinoma. Front. Genet. 12, 706814. 10.3389/fgene.2021.706814 34539740 PMC8441018

[B6] Chandel A. Goyal A. K. Ghosh G. Rath G. (2019). Recent advances in aerosolised drug delivery. Biomed. Pharmacother. 112, 108601. 10.1016/j.biopha.2019.108601 30780107

[B7] Chen C. (2004). Searching for intellectual turning points: progressive knowledge domain visualization. Proc. Natl. Acad. Sci. U S A. 101 (Suppl. 1), 5303–5310. 10.1073/pnas.0307513100 14724295 PMC387312

[B8] Chen C. Song M. (2019). Visualizing a field of research: a methodology of systematic scientometric reviews. PLoS One 14 (10), e0223994. 10.1371/journal.pone.0223994 31671124 PMC6822756

[B9] Chen C. Yang Y. Guo Y. He J. Chen Z. Qiu S. (2023c). CYP1B1 inhibits ferroptosis and induces anti-PD-1 resistance by degrading ACSL4 in colorectal cancer. Cell Death Dis. 14 (4), 271. 10.1038/s41419-023-05803-2 37059712 PMC10104818

[B10] Chen L. Cai Q. Yang R. Wang H. Ling H. Li T. (2023a). GINS4 suppresses ferroptosis by antagonizing p53 acetylation with Snail. Proc. Natl. Acad. Sci. U S A. 120 (15), e2219585120. 10.1073/pnas.2219585120 37018198 PMC10104543

[B11] Chen Q. Zheng W. Guan J. Liu H. Dan Y. Zhu L. (2023b). SOCS2-enhanced ubiquitination of SLC7A11 promotes ferroptosis and radiosensitization in hepatocellular carcinoma. Cell Death Differ. 30 (1), 137–151. 10.1038/s41418-022-01051-7 35995846 PMC9883449

[B12] Chen X. Li J. Kang R. Klionsky D. J. Tang D. (2021). Ferroptosis: machinery and regulation. Autophagy 17 (9), 2054–2081. 10.1080/15548627.2020.1810918 32804006 PMC8496712

[B13] Conche C. Finkelmeier F. Pešić M. Nicolas A. M. Böttger T. W. Kennel K. B. (2023). Combining ferroptosis induction with MDSC blockade renders primary tumours and metastases in liver sensitive to immune checkpoint blockade. Gut 72 (9), 1774–1782. 10.1136/gutjnl-2022-327909 36707233 PMC10423492

[B14] Dai E. Chen X. Linkermann A. Jiang X. Kang R. Kagan V. E. (2024). A guideline on the molecular ecosystem regulating ferroptosis. Nat. Cell Biol. 26 (9), 1447–1457. 10.1038/s41556-024-01360-8 38424270 PMC11650678

[B15] Denton A. E. Roberts E. W. Fearon D. T. (2018). Stromal cells in the tumor microenvironment. Adv. Exp. Med. Biol. 1060, 99–114. 10.1007/978-3-319-78127-3_6 30155624

[B16] Ding Y. Chen X. Liu C. Ge W. Wang Q. Hao X. (2021). Identification of a small molecule as inducer of ferroptosis and apoptosis through ubiquitination of GPX4 in triple negative breast cancer cells. J. Hematol. Oncol. 14 (1), 19. 10.1186/s13045-020-01016-8 33472669 PMC7816340

[B17] Dixon S. J. Lemberg K. M. Lamprecht M. R. Skouta R. Zaitsev E. M. Gleason C. E. (2012). Ferroptosis: an iron-dependent form of nonapoptotic cell death. Cell 149 (5), 1060–1072. 10.1016/j.cell.2012.03.042 22632970 PMC3367386

[B18] Dixon S. J. Olzmann J. A. (2024). The cell biology of ferroptosis. Nat. Rev. Mol. Cell Biol. 25, 424–442. 10.1038/s41580-024-00703-5 38366038 PMC12187608

[B19] Doll S. Freitas F. P. Shah R. Aldrovandi M. da Silva M. C. Ingold I. (2019). FSP1 is a glutathione-independent ferroptosis suppressor. Nature 575 (7784), 693–698. 10.1038/s41586-019-1707-0 31634899

[B20] Doll S. Proneth B. Tyurina Y. Y. Panzilius E. Kobayashi S. Ingold I. (2017). ACSL4 dictates ferroptosis sensitivity by shaping cellular lipid composition. Nat. Chem. Biol. 13 (1), 91–98. 10.1038/nchembio.2239 27842070 PMC5610546

[B21] Fan J. Lin H. Luo J. Chen L. (2025). 4-Methoxydalbergione inhibits the tumorigenesis and metastasis of lung cancer through promoting ferroptosis via the DNMT1/system Xc-/GPX4 pathway. Mol. Med. Rep. 31 (1), 19. 10.3892/mmr.2024.13384 39513605 PMC11564907

[B22] Fu F. Wang W. Wu L. Wang W. Huang Z. Huang Y. (2023). Inhalable biomineralized liposomes for cyclic Ca(2+)-burst-centered endoplasmic reticulum stress enhanced lung cancer ferroptosis therapy. ACS Nano 17 (6), 5486–5502. 10.1021/acsnano.2c10830 36883602

[B23] Galluzzi L. Vitale I. Aaronson S. A. Abrams J. M. Adam D. Agostinis P. (2018). Molecular mechanisms of cell death: recommendations of the nomenclature committee on cell death 2018. Cell Death Differ. 25 (3), 486–541. 10.1038/s41418-017-0012-4 29362479 PMC5864239

[B24] Gao W. Wang X. Zhou Y. Wang X. Yu Y. (2022). Autophagy, ferroptosis, pyroptosis, and necroptosis in tumor immunotherapy. Signal Transduct. Target Ther. 7 (1), 196. 10.1038/s41392-022-01046-3 35725836 PMC9208265

[B25] Guo J. Xu B. Han Q. Zhou H. Xia Y. Gong C. (2018). Ferroptosis: a novel anti-tumor action for cisplatin. Cancer Res. Treat. 50 (2), 445–460. 10.4143/crt.2016.572 28494534 PMC5912137

[B26] Han N. Yang Z. Y. Xie Z. X. Xu H. Z. Yu T. T. Li Q. R. (2023). Dihydroartemisinin elicits immunogenic death through ferroptosis-triggered ER stress and DNA damage for lung cancer immunotherapy. Phytomedicine 112, 154682. 10.1016/j.phymed.2023.154682 36739636

[B27] Hassannia B. Vandenabeele P. Vanden Berghe T. (2019). Targeting ferroptosis to iron out cancer. Cancer Cell 35 (6), 830–849. 10.1016/j.ccell.2019.04.002 31105042

[B28] Hendricks J. M. Doubravsky C. E. Wehri E. Li Z. Roberts M. A. Deol K. K. (2023). Identification of structurally diverse FSP1 inhibitors that sensitize cancer cells to ferroptosis. Cell Chem. Biol. 30 (9), 1090–1103.e7. 10.1016/j.chembiol.2023.04.007 37178691 PMC10524360

[B29] Hengrui L. (2022). Toxic medicine used in Traditional Chinese Medicine for cancer treatment: are ion channels involved? J. Tradit. Chin. Med. 42 (6), 1019–1022. 10.19852/j.cnki.jtcm.20220815.005 36378062 PMC9924727

[B30] Ho L. Crabtree G. R. (2010). Chromatin remodelling during development. Nature 463 (7280), 474–484. 10.1038/nature08911 20110991 PMC3060774

[B31] Hou J. Jiang C. Wen X. Li C. Xiong S. Yue T. (2022). ACSL4 as a potential target and biomarker for anticancer: from molecular mechanisms to clinical therapeutics. Front. Pharmacol. 13, 949863. 10.3389/fphar.2022.949863 35910359 PMC9326356

[B32] Hsieh C. H. Hsieh H. C. Shih F. S. Wang P. W. Yang L. X. Shieh D. B. (2021). An innovative NRF2 nano-modulator induces lung cancer ferroptosis and elicits an immunostimulatory tumor microenvironment. Theranostics 11 (14), 7072–7091. 10.7150/thno.57803 34093872 PMC8171079

[B33] Huang F. Pang J. Xu L. Niu W. Zhang Y. Li S. (2022). Hedyotis diffusa injection induces ferroptosis via the Bax/Bcl2/VDAC2/3 axis in lung adenocarcinoma. Phytomedicine 104, 154319. 10.1016/j.phymed.2022.154319 35853302

[B34] Jelic M. D. Mandic A. D. Maricic S. M. Srdjenovic B. U. (2021). Oxidative stress and its role in cancer. J. Cancer Res. Ther. 17 (1), 22–28. 10.4103/jcrt.JCRT_862_16 33723127

[B35] Jiang X. Stockwell B. R. Conrad M. (2021). Ferroptosis: mechanisms, biology and role in disease. Nat. Rev. Mol. Cell Biol. 22 (4), 266–282. 10.1038/s41580-020-00324-8 33495651 PMC8142022

[B36] Jiang Y. Mao C. Yang R. Yan B. Shi Y. Liu X. (2017). EGLN1/c-Myc induced lymphoid-specific helicase inhibits ferroptosis through lipid metabolic gene expression changes. Theranostics 7 (13), 3293–3305. 10.7150/thno.19988 28900510 PMC5595132

[B37] Jiao Y. Liu X. Shi J. An J. Yu T. Zou G. (2024). Unraveling the interplay of ferroptosis and immune dysregulation in diabetic kidney disease: a comprehensive molecular analysis. Diabetol. Metab. Syndr. 16 (1), 86. 10.1186/s13098-024-01316-w 38643193 PMC11032000

[B38] Kim J. W. Min D. W. Kim D. Kim J. Kim M. J. Lim H. (2023). GPX4 overexpressed non-small cell lung cancer cells are sensitive to RSL3-induced ferroptosis. Sci. Rep. 13 (1), 8872. 10.1038/s41598-023-35978-9 37258589 PMC10232506

[B39] Kinowaki Y. Taguchi T. Onishi I. Kirimura S. Kitagawa M. Yamamoto K. (2021). Overview of ferroptosis and synthetic lethality strategies. Int. J. Mol. Sci. 22 (17), 9271. 10.3390/ijms22179271 34502181 PMC8430824

[B40] Koppula P. Lei G. Zhang Y. Yan Y. Mao C. Kondiparthi L. (2022). A targetable CoQ-FSP1 axis drives ferroptosis- and radiation-resistance in KEAP1 inactive lung cancers. Nat. Commun. 13 (1), 2206. 10.1038/s41467-022-29905-1 35459868 PMC9033817

[B41] Koppula P. Zhuang L. Gan B. (2021). Cystine transporter SLC7A11/xCT in cancer: ferroptosis, nutrient dependency, and cancer therapy. Protein Cell 12 (8), 599–620. 10.1007/s13238-020-00789-5 33000412 PMC8310547

[B42] Kuwata H. Hara S. (2019). Role of acyl-CoA synthetase ACSL4 in arachidonic acid metabolism. Prostagl. Other Lipid Mediat 144, 106363. 10.1016/j.prostaglandins.2019.106363 31306767

[B43] Lai Y. Zhang Z. Li J. Li W. Huang Z. Zhang C. (2019). STYK1/NOK correlates with ferroptosis in non-small cell lung carcinoma. Biochem. Biophys. Res. Commun. 519 (4), 659–666. 10.1016/j.bbrc.2019.09.032 31542233

[B44] Lei G. Zhuang L. Gan B. (2022). Targeting ferroptosis as a vulnerability in cancer. Nat. Rev. Cancer 22 (7), 381–396. 10.1038/s41568-022-00459-0 35338310 PMC10243716

[B45] Li W. Wu H. Xu J. (2023a). Construction of a genomic instability-derived predictive prognostic signature for non-small cell lung cancer patients. Cancer Genet. 278-279, 24–37. 10.1016/j.cancergen.2023.07.008 37579716

[B46] Li X. Zhu S. Li Z. Meng Y. Q. Huang S. J. Yu Q. Y. (2022). Melittin induces ferroptosis and ER stress-CHOP-mediated apoptosis in A549 cells. Free Radic. Res. 56 (5-6), 398–410. 10.1080/10715762.2022.2131551 36194238

[B47] Li Y. Yang W. Zheng Y. Dai W. Ji J. Wu L. (2023b). Targeting fatty acid synthase modulates sensitivity of hepatocellular carcinoma to sorafenib via ferroptosis. J. Exp. Clin. Cancer Res. 42 (1), 6. 10.1186/s13046-022-02567-z 36604718 PMC9817350

[B48] Liu H. (2023). Expression and potential immune involvement of cuproptosis in kidney renal clear cell carcinoma. Cancer Genet. 274-275, 21–25. 10.1016/j.cancergen.2023.03.002 36963335

[B49] Liu H. Tang T. (2022a). Pan-cancer genetic analysis of cuproptosis and copper metabolism-related gene set. Front. Oncol. 12, 952290. 10.3389/fonc.2022.952290 36276096 PMC9582932

[B50] Liu H. Tang T. (2022b). Pan-cancer genetic analysis of cuproptosis and copper metabolism-related gene set. Am. J. Cancer Res. 12 (8), 4074–4081. 10.3389/fonc.2022.952290 36276096 PMC9582932

[B51] Liu H. Tang T. (2023). Pan-cancer genetic analysis of disulfidptosis-related gene set. Cancer Genet. 278-279, 91–103. 10.1016/j.cancergen.2023.10.001 37879141

[B52] Liu T. Yang Q. Zheng H. Jia H. He Y. Zhang X. (2021). Multifaceted roles of a bioengineered nanoreactor in repressing radiation-induced lung injury. Biomaterials 277, 121103. 10.1016/j.biomaterials.2021.121103 34478930

[B53] Liu X. Nie L. Zhang Y. Yan Y. Wang C. Colic M. (2023a). Actin cytoskeleton vulnerability to disulfide stress mediates disulfidptosis. Nat. Cell Biol. 25 (3), 404–414. 10.1038/s41556-023-01091-2 36747082 PMC10027392

[B54] Liu Y. Wan Y. Jiang Y. Zhang L. Cheng W. (2023b). GPX4: the hub of lipid oxidation, ferroptosis, disease and treatment. Biochim. Biophys. Acta Rev. Cancer 1878 (3), 188890. 10.1016/j.bbcan.2023.188890 37001616

[B55] Long Y. Guo J. Chen J. Sun J. Wang H. Peng X. (2023). GPR162 activates STING dependent DNA damage pathway as a novel tumor suppressor and radiation sensitizer. Signal Transduct. Target Ther. 8 (1), 48. 10.1038/s41392-022-01224-3 36725837 PMC9892510

[B56] Lou J. S. Zhao L. P. Huang Z. H. Chen X. Y. Xu J. T. Tai W. C. (2021). Ginkgetin derived from Ginkgo biloba leaves enhances the therapeutic effect of cisplatin via ferroptosis-mediated disruption of the Nrf2/HO-1 axis in EGFR wild-type non-small-cell lung cancer. Phytomedicine 80, 153370. 10.1016/j.phymed.2020.153370 33113504

[B57] Lugano R. Ramachandran M. Dimberg A. (2020). Tumor angiogenesis: causes, consequences, challenges and opportunities. Cell Mol. Life Sci. 77 (9), 1745–1770. 10.1007/s00018-019-03351-7 31690961 PMC7190605

[B58] Ma L. Chen T. Zhang X. Miao Y. Tian X. Yu K. (2021a). The m(6)A reader YTHDC2 inhibits lung adenocarcinoma tumorigenesis by suppressing SLC7A11-dependent antioxidant function. Redox Biol. 38, 101801. 10.1016/j.redox.2020.101801 33232910 PMC7691619

[B59] Ma L. Zhang X. Yu K. Xu X. Chen T. Shi Y. (2021b). Targeting SLC3A2 subunit of system X(C)(-) is essential for m(6)A reader YTHDC2 to be an endogenous ferroptosis inducer in lung adenocarcinoma. Free Radic. Biol. Med. 168, 25–43. 10.1016/j.freeradbiomed.2021.03.023 33785413

[B60] Ma L. F. Huang H. Feng W. Chen L. Xia L. L. Yu Y. C. (2022). 2D catalytic, chemodynamic, and ferroptotic vermiculite nanomedicine. Adv. Funct. Mater. 32 (51), 16. 10.1002/adfm.202208220

[B61] Ma Z. Liu D. Li W. Di S. Zhang Z. Zhang J. (2019). STYK1 promotes tumor growth and metastasis by reducing SPINT2/HAI-2 expression in non-small cell lung cancer. Cell Death Dis. 10 (6), 435. 10.1038/s41419-019-1659-1 31164631 PMC6547759

[B62] Mao C. Wang X. Liu Y. Wang M. Yan B. Jiang Y. (2018). A G3BP1-interacting lncRNA promotes ferroptosis and apoptosis in cancer via nuclear sequestration of p53. Cancer Res. 78 (13), 3484–3496. 10.1158/0008-5472.Can-17-3454 29588351 PMC8073197

[B63] Mao W. Cai Y. Chen D. Jiang G. Xu Y. Chen R. (2022). Statin shapes inflamed tumor microenvironment and enhances immune checkpoint blockade in non-small cell lung cancer. JCI Insight 7 (18), e161940. 10.1172/jci.insight.161940 35943796 PMC9675559

[B64] Ni J. Chen K. Zhang J. Zhang X. (2021). Inhibition of GPX4 or mTOR overcomes resistance to Lapatinib via promoting ferroptosis in NSCLC cells. Biochem. Biophys. Res. Commun. 567, 154–160. 10.1016/j.bbrc.2021.06.051 34157442

[B65] Niu X. Chen L. Li Y. Hu Z. He F. (2022). Ferroptosis, necroptosis, and pyroptosis in the tumor microenvironment: perspectives for immunotherapy of SCLC. Semin. Cancer Biol. 86 (Pt 3), 273–285. 10.1016/j.semcancer.2022.03.009 35288298

[B66] Pan X. Lin Z. Jiang D. Yu Y. Yang D. Zhou H. (2019). Erastin decreases radioresistance of NSCLC cells partially by inducing GPX4-mediated ferroptosis. Oncol. Lett. 17 (3), 3001–3008. 10.3892/ol.2019.9888 30854078 PMC6365906

[B67] Panda S. K. Peng V. Sudan R. Ulezko Antonova A. Di Luccia B. Ohara T. E. (2023). Repression of the aryl-hydrocarbon receptor prevents oxidative stress and ferroptosis of intestinal intraepithelial lymphocytes. Immunity 56 (4), 797–812.e4. 10.1016/j.immuni.2023.01.023 36801011 PMC10101911

[B68] Peng Y. Ouyang L. Zhou Y. Lai W. Chen Y. Wang Z. (2023). AhR promotes the development of non-small cell lung cancer by inducing SLC7A11-dependent antioxidant function. J. Cancer 14 (5), 821–834. 10.7150/jca.82066 37056388 PMC10088881

[B69] Pope L. E. Dixon S. J. (2023). Regulation of ferroptosis by lipid metabolism. Trends Cell Biol. 33 (12), 1077–1087. 10.1016/j.tcb.2023.05.003 37407304 PMC10733748

[B70] Reychler G. Michotte J. B. (2019). Development challenges and opportunities in aerosol drug delivery systems in non-invasive ventilation in adults. Expert Opin. Drug Deliv. 16 (2), 153–162. 10.1080/17425247.2019.1572111 30658045

[B71] Shu L. Luo P. Chen Q. Liu J. Huang Y. Wu C. (2024). Fibroin nanodisruptor with Ferroptosis-Autophagy synergism is potent for lung cancer treatment. Int. J. Pharm. 664, 124582. 10.1016/j.ijpharm.2024.124582 39142466

[B72] Sicard G. Protzenko D. Giacometti S. Barlési F. Ciccolini J. Fanciullino R. (2024). Overcoming immuno-resistance by rescheduling anti-VEGF/cytotoxics/anti-PD-1 combination in lung cancer model. Cancer Drug Resist 7, 10. 10.20517/cdr.2023.146 38510749 PMC10951825

[B73] Siegel R. L. Miller K. D. Wagle N. S. Jemal A. (2023). Cancer statistics, 2023. CA Cancer J. Clin. 73 (1), 17–48. 10.3322/caac.21763 36633525

[B74] Singh S. Goyal D. Raman K. Kumar S. Malik P. S. Elangovan R. (2023). RNA profile of immuno-magnetically enriched lung cancer associated exosomes isolated from clinical samples. Cancer Genet. 274-275, 59–71. 10.1016/j.cancergen.2023.03.008 37030018

[B75] Song X. Long D. (2020). Nrf2 and ferroptosis: a new research direction for neurodegenerative diseases. Front. Neurosci. 14, 267. 10.3389/fnins.2020.00267 32372896 PMC7186402

[B76] Stockwell B. R. (2022). Ferroptosis turns 10: emerging mechanisms, physiological functions, and therapeutic applications. Cell 185 (14), 2401–2421. 10.1016/j.cell.2022.06.003 35803244 PMC9273022

[B77] Stockwell B. R. Friedmann Angeli J. P. Bayir H. Bush A. I. Conrad M. Dixon S. J. (2017). Ferroptosis: a regulated cell death nexus linking metabolism, redox biology, and disease. Cell 171 (2), 273–285. 10.1016/j.cell.2017.09.021 28985560 PMC5685180

[B78] Sun X. Niu X. Chen R. He W. Chen D. Kang R. (2016). Metallothionein-1G facilitates sorafenib resistance through inhibition of ferroptosis. Hepatology 64 (2), 488–500. 10.1002/hep.28574 27015352 PMC4956496

[B79] Sung H. Ferlay J. Siegel R. L. Laversanne M. Soerjomataram I. Jemal A. (2021). Global cancer statistics 2020: GLOBOCAN estimates of incidence and mortality worldwide for 36 cancers in 185 countries. CA Cancer J. Clin. 71 (3), 209–249. 10.3322/caac.21660 33538338

[B80] Takahashi N. Cho P. Selfors L. M. Kuiken H. J. Kaul R. Fujiwara T. (2020). 3D culture models with CRISPR screens reveal hyperactive NRF2 as a prerequisite for spheroid formation via regulation of proliferation and ferroptosis. Mol. Cell 80 (5), 828–844.e6. 10.1016/j.molcel.2020.10.010 33128871 PMC7718371

[B81] Tang J. Long G. Hu K. Xiao D. Liu S. Xiao L. (2023). Targeting USP8 inhibits O-GlcNAcylation of SLC7A11 to promote ferroptosis of hepatocellular carcinoma via stabilization of OGT. Adv. Sci. (Weinh) 10 (33), e2302953. 10.1002/advs.202302953 37867237 PMC10667802

[B82] Tang X. Ding H. Liang M. Chen X. Yan Y. Wan N. (2021). Curcumin induces ferroptosis in non-small-cell lung cancer via activating autophagy. Thorac. Cancer 12 (8), 1219–1230. 10.1111/1759-7714.13904 33656766 PMC8046146

[B83] Thai A. A. Solomon B. J. Sequist L. V. Gainor J. F. Heist R. S. (2021). Lung cancer. Lancet 398 (10299), 535–554. 10.1016/s0140-6736(21)00312-3 34273294

[B84] Tu Y. (2016). Artemisinin-A gift from traditional Chinese medicine to the world (nobel lecture). Angew. Chem. Int. Ed. Engl. 55 (35), 10210–10226. 10.1002/anie.201601967 27488942

[B85] Wang J. Wong Y. K. Liao F. (2018). What has traditional Chinese medicine delivered for modern medicine? Expert Rev. Mol. Med. 20, e4. 10.1017/erm.2018.3 29747718

[B86] Wang J. Yang W. He X. Zhang Z. Zheng X. (2022c). Assembling p53 activating peptide with CeO(2) nanoparticle to construct a metallo-organic supermolecule toward the synergistic ferroptosis of tumor. Front. Bioeng. Biotechnol. 10, 929536. 10.3389/fbioe.2022.929536 35837547 PMC9273839

[B87] Wang M. Mao C. Ouyang L. Liu Y. Lai W. Liu N. (2019a). Long noncoding RNA LINC00336 inhibits ferroptosis in lung cancer by functioning as a competing endogenous RNA. Cell Death Differ. 26 (11), 2329–2343. 10.1038/s41418-019-0304-y 30787392 PMC6889193

[B88] Wang W. Green M. Choi J. E. Gijón M. Kennedy P. D. Johnson J. K. (2019b). CD8(+) T cells regulate tumour ferroptosis during cancer immunotherapy. Nature 569 (7755), 270–274. 10.1038/s41586-019-1170-y 31043744 PMC6533917

[B89] Wang W. Zhang J. Wang Y. Xu Y. Zhang S. (2022b). Identifies microtubule-binding protein CSPP1 as a novel cancer biomarker associated with ferroptosis and tumor microenvironment. Comput. Struct. Biotechnol. J. 20, 3322–3335. 10.1016/j.csbj.2022.06.046 35832625 PMC9253833

[B90] Wang X. Chen Y. Wang X. Tian H. Wang Y. Jin J. (2021c). Stem cell factor SOX2 confers ferroptosis resistance in lung cancer via upregulation of SLC7A11. Cancer Res. 81 (20), 5217–5229. 10.1158/0008-5472.Can-21-0567 34385181 PMC8530936

[B91] Wang Y. Chen Z. Luo J. Zhang J. Sang A. M. Cheng Z. S. (2023). Salidroside postconditioning attenuates ferroptosis-mediated lung ischemia-reperfusion injury by activating the Nrf2/SLC7A11 signaling axis. Int. Immunopharmacol. 115, 109731. 10.1016/j.intimp.2023.109731 36907990

[B92] Wang Y. Li D. Jia Z. Hui J. Xin Q. Zhou Q. (2022a). A bibliometric analysis of research on the links between gut microbiota and atherosclerosis. Front. Cardiovasc Med. 9, 941607. 10.3389/fcvm.2022.941607 35903667 PMC9314574

[B93] Wang Y. Qiu S. Wang H. Cui J. Tian X. Miao Y. (2021b). Transcriptional repression of ferritin light chain increases ferroptosis sensitivity in lung adenocarcinoma. Front. Cell Dev. Biol. 9, 719187. 10.3389/fcell.2021.719187 34765600 PMC8576304

[B94] Wang Y. Yang L. Zhang X. Cui W. Liu Y. Sun Q. R. (2019c). Epigenetic regulation of ferroptosis by H2B monoubiquitination and p53. EMBO Rep. 20 (7), e47563. 10.15252/embr.201847563 31267712 PMC6607012

[B95] Wang Z. Zhang X. Tian X. Yang Y. Ma L. Wang J. (2021a). CREB stimulates GPX4 transcription to inhibit ferroptosis in lung adenocarcinoma. Oncol. Rep. 45 (6), 88. 10.3892/or.2021.8039 33846793 PMC8042667

[B96] Wen R. J. Dong X. Zhuang H. W. Pang F. X. Ding S. C. Li N. (2023). Baicalin induces ferroptosis in osteosarcomas through a novel Nrf2/xCT/GPX4 regulatory axis. Phytomedicine 116, 154881. 10.1016/j.phymed.2023.154881 37209607

[B97] Wu J. Minikes A. M. Gao M. Bian H. Li Y. Stockwell B. R. (2019). Intercellular interaction dictates cancer cell ferroptosis via NF2-YAP signalling. Nature 572 (7769), 402–406. 10.1038/s41586-019-1426-6 31341276 PMC6697195

[B98] Wu M. Zhang X. Zhang W. Chiou Y. S. Qian W. Liu X. (2022a). Cancer stem cell regulated phenotypic plasticity protects metastasized cancer cells from ferroptosis. Nat. Commun. 13 (1), 1371. 10.1038/s41467-022-29018-9 35296660 PMC8927306

[B99] Wu Q. Chen Z. Ding Y. Tang Y. Cheng Y. (2022b). Protective effect of traditional Chinese medicine on non-alcoholic fatty liver disease and liver cancer by targeting ferroptosis. Front. Nutr. 9, 1033129. 10.3389/fnut.2022.1033129 36330148 PMC9623008

[B100] Wu S. Zhu C. Tang D. Dou Q. P. Shen J. Chen X. (2021). The role of ferroptosis in lung cancer. Biomark. Res. 9 (1), 82. 10.1186/s40364-021-00338-0 34742351 PMC8572460

[B101] Wu X. Sui Z. Zhang H. Wang Y. Yu Z. (2020). Integrated analysis of lncRNA-mediated ceRNA network in lung adenocarcinoma. Front. Oncol. 10, 554759. 10.3389/fonc.2020.554759 33042838 PMC7523091

[B102] Xia X. Fan X. Zhao M. Zhu P. (2019). The relationship between ferroptosis and tumors: a novel landscape for therapeutic approach. Curr. Gene Ther. 19 (2), 117–124. 10.2174/1566523219666190628152137 31264548 PMC7046989

[B103] Xian Z. Y. Hu B. Wang T. Cai J. L. Zeng J. Y. Zou Q. (2020). CircABCB10 silencing inhibits the cell ferroptosis and apoptosis by regulating the miR-326/CCL5 axis in rectal cancer. Neoplasma 67 (5), 1063–1073. 10.4149/neo_2020_191024N1084 32567935

[B104] Xing N. Du Q. Guo S. Xiang G. Zhang Y. Meng X. (2023). Ferroptosis in lung cancer: a novel pathway regulating cell death and a promising target for drug therapy. Cell Death Discov. 9 (1), 110. 10.1038/s41420-023-01407-z 37005430 PMC10067943

[B105] Xu X. Cui J. Wang H. Ma L. Zhang X. Guo W. (2022). IGF2BP3 is an essential N(6)-methyladenosine biotarget for suppressing ferroptosis in lung adenocarcinoma cells. Mater Today Bio 17, 100503. 10.1016/j.mtbio.2022.100503 PMC9707255 36457846

[B106] Xue X. Ma L. Zhang X. Xu X. Guo S. Wang Y. (2022). Tumour cells are sensitised to ferroptosis via RB1CC1-mediated transcriptional reprogramming. Clin. Transl. Med. 12 (2), e747. 10.1002/ctm2.747 35220675 PMC8882240

[B107] Yang R. Liu N. Chen L. Jiang Y. Shi Y. Mao C. (2019). LSH interacts with and stabilizes GINS4 transcript that promotes tumourigenesis in non-small cell lung cancer. J. Exp. Clin. Cancer Res. 38 (1), 280. 10.1186/s13046-019-1276-y 31253190 PMC6599244

[B108] Ye Y. Chen A. Li L. Liang Q. Wang S. Dong Q. (2022). Repression of the antiporter SLC7A11/glutathione/glutathione peroxidase 4 axis drives ferroptosis of vascular smooth muscle cells to facilitate vascular calcification. Kidney Int. 102 (6), 1259–1275. 10.1016/j.kint.2022.07.034 36063875

[B109] Zhang C. Wang C. Yang Z. Bai Y. Shukuya T. Poh M. E. (2022b). Identification of GPX4 as a therapeutic target for lung adenocarcinoma after EGFR-TKI resistance. Transl. Lung Cancer Res. 11 (5), 786–801. 10.21037/tlcr-22-318 35693278 PMC9186177

[B110] Zhang H. Deng T. Liu R. Ning T. Yang H. Liu D. (2020). CAF secreted miR-522 suppresses ferroptosis and promotes acquired chemo-resistance in gastric cancer. Mol. Cancer 19 (1), 43. 10.1186/s12943-020-01168-8 32106859 PMC7045485

[B111] Zhang J. Song L. Xu L. Fan Y. Wang T. Tian W. (2021a). Knowledge domain and emerging trends in ferroptosis research: a bibliometric and knowledge-map analysis. Front. Oncol. 11, 686726. 10.3389/fonc.2021.686726 34150654 PMC8209495

[B112] Zhang M. M. Gong Z. C. Chen Y. Y. (2023). Research progress on oxaliplatin-induced neurotoxicity in traditional Chinese medicine (TCM) and western medical cognition and prevention and treatment by TCM. Zhongguo Zhong Yao Za Zhi 48 (17), 4610–4619. 10.19540/j.cnki.cjcmm.20230605.601 37802800

[B113] Zhang W. Jiang B. Liu Y. Xu L. Wan M. (2022a). Bufotalin induces ferroptosis in non-small cell lung cancer cells by facilitating the ubiquitination and degradation of GPX4. Free Radic. Biol. Med. 180, 75–84. 10.1016/j.freeradbiomed.2022.01.009 35038550

[B114] Zhang W. Yao S. Huang H. Zhou H. Zhou H. Wei Q. (2021b). Molecular subtypes based on ferroptosis-related genes and tumor microenvironment infiltration characterization in lung adenocarcinoma. Oncoimmunology 10 (1), 1959977. 10.1080/2162402x.2021.1959977 34527427 PMC8437492

[B115] Zheng Y. Li L. Chen H. Zheng Y. Tan X. Zhang G. (2023). Luteolin exhibits synergistic therapeutic efficacy with erastin to induce ferroptosis in colon cancer cells through the HIC1-mediated inhibition of GPX4 expression. Free Radic. Biol. Med. 208, 530–544. 10.1016/j.freeradbiomed.2023.09.014 37717793

[B116] Zou J. Wang L. Tang H. Liu X. Peng F. Peng C. (2021). Ferroptosis in non-small cell lung cancer: progression and therapeutic potential on it. Int. J. Mol. Sci. 22 (24), 13335. 10.3390/ijms222413335 34948133 PMC8704137

[B117] Zukotynski K. A. Hasan O. K. Lubanovic M. Gerbaudo V. H. (2021). Update on molecular imaging and precision medicine in lung cancer. Radiol. Clin. North Am. 59 (5), 693–703. 10.1016/j.rcl.2021.05.002 34392913

[B118] Zuo Y. B. Zhang Y. F. Zhang R. Tian J. W. Lv X. B. Li R. (2022). Ferroptosis in cancer progression: role of noncoding RNAs. Int. J. Biol. Sci. 18 (5), 1829–1843. 10.7150/ijbs.66917 35342359 PMC8935228

